# Evolution of specifier proteins in glucosinolate-containing plants

**DOI:** 10.1186/1471-2148-12-127

**Published:** 2012-07-28

**Authors:** Jennifer C Kuchernig, Meike Burow, Ute Wittstock

**Affiliations:** 1 Institute of Pharmaceutical Biology, Technische Universität Braunschweig, Mendelssohnstr. 1, D-38106 Braunschweig, Germany; 2 DynaMo Centre of Excellence and VKR Research Centre for Pro-Active Plants, Department of Plant and Environmental Sciences, Faculty of Science, University of Copenhagen, Thorvaldsensvej 40, 1871, Frederiksberg C, Denmark

**Keywords:** Specifier proteins, Glucosinolate breakdown, Chemical diversity, Phylogenetic analysis, Secondary metabolism

## Abstract

**Background:**

The glucosinolate-myrosinase system is an activated chemical defense system found in plants of the Brassicales order. Glucosinolates are stored separately from their hydrolytic enzymes, the myrosinases, in plant tissues. Upon tissue damage, e.g. by herbivory, glucosinolates and myrosinases get mixed and glucosinolates are broken down to an array of biologically active compounds of which isothiocyanates are toxic to a wide range of organisms. Specifier proteins occur in some, but not all glucosinolate-containing plants and promote the formation of biologically active non-isothiocyanate products upon myrosinase-catalyzed glucosinolate breakdown.

**Results:**

Based on a phytochemical screening among representatives of the Brassicales order, we selected candidate species for identification of specifier protein cDNAs. We identified ten specifier proteins from a range of species of the Brassicaceae and assigned each of them to one of the three specifier protein types (NSP, nitrile-specifier protein, ESP, epithiospecifier protein, TFP, thiocyanate-forming protein) after heterologous expression in *Escherichia coli*. Together with nine known specifier proteins and three putative specifier proteins found in databases, we subjected the newly identified specifier proteins to phylogenetic analyses. Specifier proteins formed three major clusters, named AtNSP5-cluster, AtNSP1-cluster, and ESP/TFP cluster. Within the ESP/TFP cluster, specifier proteins grouped according to the Brassicaceae lineage they were identified from. Non-synonymous vs. synonymous substitution rate ratios suggested purifying selection to act on specifier protein genes.

**Conclusions:**

Among specifier proteins, NSPs represent the ancestral activity. The data support a monophyletic origin of ESPs from NSPs. The split between NSPs and ESPs/TFPs happened before the radiation of the core Brassicaceae. Future analyses have to show if TFP activity evolved from ESPs at least twice independently in different Brassicaceae lineages as suggested by the phylogeny. The ability to form non-isothiocyanate products by specifier protein activity may provide plants with a selective advantage. The evolution of specifier proteins in the Brassicaceae demonstrates the plasticity of secondary metabolism within an activated plant defense system.

## Background

**Figure 1:**
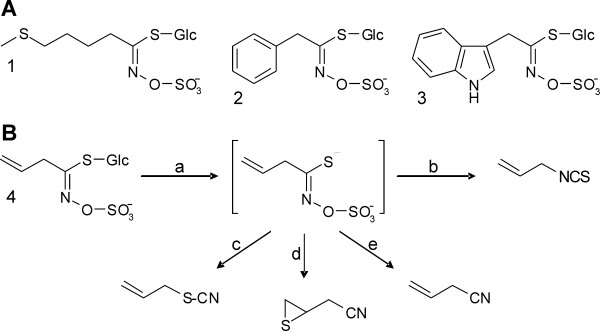
**Glucosinolates and glucosinolate hydrolysis. A**. Examples of structures of aliphatic glucosinolates (**1**, 4-methylthiobutylglucosinolate), aromatic (i.e. Phe-derived) glucosinolates (**2**, benzylglucosinolate), and indolic glucosinolates (**3**, indol-3-ylmethylglucosinolate). **B**. Using allylglucosinolate (**4**, aliphatic side chain with terminal double bond) as an example, the hydrolysis by myrosinase (**a**) is shown that leads to the formation of an isothiocyanate upon spontaneous rearrangement of the aglycone (**b**) or to the formation of alternative products (**c**-**e**). The ability to form organic thiocyanates depends on structural requirements of the glucosinolate side chain and the presence of thiocyanate-forming protein (TFP; **c**). Epithiospecifier proteins (ESPs) promote the formation of epithionitriles upon hydrolysis of glucosinolates with a terminal double bond (**d**), but do not support organic thiocyanate formation. Simple nitriles are formed at acidic pH, in the presence of ferrous ions or due to the action of nitrile-specifier proteins (NSPs; **e**). If a glucosinolate does not fullfill the structural requirements for epithionitrile formation, ESPs promote the conversion of the aglycone to the simple nitrile. In a similar way, TFPs support epithionitrile or simple nitrile formation upon myrosinase-catalyzed hydrolysis of glucosinolates that do not allow organic thiocyanate formation.

A distinguished feature of plant secondary metabolism is its enormous diversity. More than 200 000 secondary metabolites are known to date that are either widely distributed in the plant kingdom or specific for a small group of plant taxa. Evidence is accumulating that this chemical diversity is shaped by selection pressures exerted by the biotic and abiotic environment of the plant [1,2]. Diversification of secondary metabolism has been investigated in a number of plant systems, yet, we are only beginning to understand how metabolic diversity is generated and how it impacts ecological interactions [3-10]. The glucosinolate-myrosinase system represents one of the best studied plant chemical defenses. Glucosinolates are amino acid-derived thioglucosides with a sulfated aldoxime moiety and variable side chains (Figure 1A) that are present in the Brassicales [11-13]. In contrast to many other chemical defenses, the glucosinolates themselves are non-toxic. They become activated upon tissue damage when endogenous thioglucosidases, the myrosinases, are released from their separate storage compartments and hydrolyze the glucosinolates. This results in the formation of biologically active breakdown products that play a role in plant-pathogen and plant-insect interactions and have attracted a lot of interest as health-promoting compounds in our diet [14-18].

In the glucosinolate-myrosinase system, chemical diversity is generated by independent variation of glucosinolate biosynthesis and glucosinolate breakdown [5,19]. Genetic variation at glucosinolate biosynthetic loci allows the production of more than 120 different glucosinolates from only a few amino acids [13]. Structural diversity is further amplified during glucosinolate breakdown whose outcome mainly depends on the structure of the glucosinolate side chain and the presence of supplementary proteins under physiological conditions [20-24] (Figure 1B). A single glucosinolate can give rise to up to four different types of breakdown products with diverse physico-chemical and biological properties. The most intensely studied breakdown products, the isothiocyanates, are formed through a spontaneous rearrangement of the glucosinolate aglucone in the absence of supplementary proteins (Figure 1B). Glucosinolate-derived isothiocyanates are very reactive and have been shown to be toxic to bacteria, fungi, nematodes and insects (reviewed in [25]). Supplementary proteins known as specifier proteins promote the rearrangement of the aglucone released by myrosinase to products other than isothiocyanates, namely to simple nitriles, epithionitriles and organic thiocyanates, without having hydrolytic activity on glucosinolates themselves [21], reviewed in [26] (Figure 1B). The biological roles of the non-isothiocyanate products are not well established, but experimental studies indicate that they may act in direct and indirect defense [25,27].

To date, nine plant specifier proteins with different substrate and product specificities have been identified at the molecular level and characterized biochemically, namely the epithiospecifier proteins (ESPs) from *Arabidopsis thaliana*[23] and *Brassica oleracea*[28], the thiocyanate-forming proteins (TFPs) from *Lepidium sativum*[20] and *Thlaspi arvense*[29] and five nitrile-specifier proteins (NSPs) from *A. thaliana*[30]. Typically, specifier proteins of different types share 50–80% amino acid sequence identity. They do not have any significant amino acid sequence identity to other functionally characterized proteins. As a structural feature, specifier proteins are predicted to contain a series of *β*-sheets representing Kelch domains often involved in protein-protein interactions [31]. In addition, four of the five NSPs contain one or two jacalin-related lectin (JAL) domains at their N-terminus [30,32]. The impact of these domains on NSP function or regulation is not known. Previous research has identified At3g07720 as an additional NSP homolog in *A. thaliana* that did not show specifier protein activity in biochemical assays and might be an ancestor of the *A. thaliana* specifier protein family with a function outside glucosinolate metabolism [30]. Furthermore, NSPs were proposed to represent the basal activity from which ESP and TFP activities derived [30]. However, evidence for this hypothesis could not be provided due to the limited number of available specifier protein sequences.

Here, we identified specifier protein cDNAs from a range of species of the Brassicaceae that showed their ability to form non-isothiocyanate products in a phytochemical screening among representatives of the Brassicales order. As the amino acid sequence identity proved not to be sufficient for the assignment of a given specifier protein to one of the three types (NSP, ESP, TFP; [29]), we expressed the cDNAs identified in *Escherichia coli* and tested the recombinant proteins for their specificity with respect to product formation upon myrosinase-catalyzed hydrolysis of two different glucosinolate substrates. Phylogenetic analyses performed with sequences of 19 characterized and three putative specifier proteins support the previous suggestion that NSP activity is the ancestral activity among specifier proteins and suggest a monophyletic origin of ESPs from NSPs. Furthermore, we show that the split between NSPs and ESPs/TFPs happened before the radiation of the core Brassicaceae that dates back to at least 36 million years ago, respectively. Phylogenetic analyses suggest that TFP activity evolved at least twice independently in different Brassicaceae lineages.

## Results and discussion

### Phytochemical screening

To study the occurrence of non-isothiocyanate products of glucosinolate hydrolysis within species of the Brassicales, we analyzed 21 species of 14 genera of the Brassicaceae as well as seven species from five other Brassicales families for their glucosinolate and glucosinolate hydrolysis product profiles (Additional file 1: Table S1). All of the Brassicaceae species investigated are representatives of the core Brassicaceae and belong to two of three major Brassicaceae lineages (lineage I and expanded lineage II) [33]. Among the 13 tribes comprised by lineage I [33], four were represented in our screening (Lepidieae, Camelineae (e.g. *Arabidopsis*), Erysimeae, Cardamineae). Among the 22 tribes of expanded lineage II [33], plants from six tribes (Alysseae, Iberideae, Arabideae (e.g. *Arabis*), Brassiceae (e.g. *Schouwia*), Isatideae, Thlaspideae) were included. Within the Brassicaceae, we identified twelve species of nine genera with non-isothiocyanate products (oxazolidine-2-thiones are isothiocyanate-derivatives and were therefore not counted as non-isothiocyanate products). Among these, eleven species produced simple nitriles, six species produced epithionitriles, and one species produced organic thiocyanate upon tissue disruption. Outside the Brassicaceae, only *Tropaeolum minus* (Tropaeolaceae) produced minor amounts of a simple nitrile (phenylacetonitrile) (Additional file 2: Figure S1). In most cases, we were able to analyze different organs or developmental stages of the plants to be screened, e.g. seedlings, leaves, roots and seeds. However, we cannot exclude that different glucosinolates and glucosinolate hydrolysis products are formed in organs or developmental stages that have not been investigated. Furthermore, environmental conditions that have not been considered here may induce biosynthetic enzymes and those involved in glucosinolate breakdown. Despite these limitations, it appears that the generation of non-isothiocyanate products is widespread in the Brassicaceae, but not common in plants of other families of the Brassicales.

### Identification and characterization of specifier protein cDNAs

Brassicaceae species that produced simple nitriles, epithionitriles or organic thiocyanates were used to identify specifier protein cDNAs by a PCR approach (with the exception of *Alyssum alpestre* due to lack of plant material). An amino acid sequence comparison of known specifier proteins identified regions of high amino acid sequence identity within NSP or ESP/TFP sequences, respectively (Additional file 3: Figure S2). As nucleotide sequence identities of the corresponding cDNAs in these regions were very high, degenerate primers were designed based on nucleotide sequences in these regions keeping a low level of degeneracy (Additional file 4: Table S2). cDNA was synthesized from total RNA isolated from leaves of species that were able to produce simple nitriles, epithionitriles or organic thiocyanates in tissue homogenates (with the exception *Schouwia purpurea* of which seedlings were used and *Alliaria petiolata* of which cotyledons were used). PCR with primers P1 and P2 using cDNA as template typically resulted in the amplification of fragments of 700–750 bp with highest similarity to *ESP/TFP* cDNAs. When primers P5 and P6 were used instead, PCR fragments of 650–700 bp were obtained that usually had highest similarity to *NSP* cDNAs. Full-length cDNAs including 3'-UTRs and at least partial 5'-UTRs were obtained by 3'- and 5'-RACE. Open reading frames (ORFs) were confirmed by independent PCR amplification with gene-specific primers using cDNA as template. ORFs were transferred to modified pET52(b) + expression vectors [29] allowing heterologous expression in *E. coli* with an N-terminal Strep-Tag II. Crude extracts of bacteria harbouring the expression constructs were subjected to specifier protein assays using allylglucosinolate and benzylglucosinolate as substrates for myrosinase (Additional file 5: Figure S3). Proteins that were able to promote the formation of organic thiocyanate from allyl- or benzylglucosinolate were designated TFPs, proteins that promoted epithionitrile formation, but not organic thiocyanate formation were designated ESPs and proteins that promoted simple nitrile, but not epithionitrile or organic thiocyanate formation from the substrates used were designated NSPs. Through this approach, ten previously unknown specifier proteins were identified, namely one TFP, six ESPs and three NSPs (Tables 1, 2). As structural features, the deduced amino acid sequences of all newly identified specifier proteins contained several copies of the kelch motif (Table 1). In addition, all NSPs identified possessed one jacaline-related lectin domain (Table 1).

**Table 1:** Characterized specifier proteins subjected to phylogenetic analyses

**Protein**	**Source**	**ORF (bp)**	**JAL-domains**	**Kelch-domains**	**Reference/ GenBank**
ApTFP1	*Alliaria petiolata*	1047	-	4	JX313341
AtESP	*Arabidopsis thaliana*	1026	-	4	[23]
AtNSP1 (At3g16400)	*Arabidopsis thaliana*	1413	1	4	[30,34]
AtNSP2 (At2g33070)	*Arabidopsis thaliana*	1416	1	4	[30,34]
AtNSP3 (At3g16390)	*Arabidopsis thaliana*	1404	1	4	[30,34]
AtNSP4 (At3g16410)	*Arabidopsis thaliana*	1860	2	4	[30,34]
AtNSP5 (At5g48180)	*Arabidopsis thaliana*	981	-	3	[30,34]
BoESP	*Brassica oleraceae*	1032	-	4	[28]
ChESP1	*Cardamine hirsuta*	1038	-	4	JX313342
ChNSP1	*Cardamine hirsuta*	1410	1	4	JX313348
CiESP1	*Cardamine impatiens*	1026	-	4	JX313343
DaESP1	*Draba aurea*	1023	-	4	JX313344
DlESP1	*Draba lanceolata*	1023	-	4	JX313345
ItESP1	*Isatis tinctoria*	1032	-	4	JX313346
ItNSP1	*Isatis tinctoria*	1410	1	4	JX313349
LsTFP	*Lepidium sativum*	1014	-	4	[20]
SpESP1	*Schouwia purpurea*	1032	-	4	JX313347
SpNSP1	*Schouwia purpurea*	1404	1	4	JX313350
TaTFP	*Thlaspi arvense*	1047	-	4	[29]

Listed are specifier proteins that have been identified and characterized biochemically in previous research or in the present study. In the latter case, GenBank accession numbers are given. For the *A. thaliana *NSPs, locus names are given in addition to the protein abbreviations as the nomenclature in the literature is not uniform. Domain structures are given according to InterProScan predictions.

**Table 2:** Activity of newly identified specifier proteins

	**Incubation with myrosinase and allylglucosinolate**			**Incubation with myrosinase and benzylglucosinolate**	
	**Thiocyanate**	**Epithionitrile**	**Simple nitrile**	**Thiocyanate**	**Simple nitrile**
ApTFP1	+	+	-	-	+
ChESP1	-	+	-	-	+
CiESP1	-	+	-	-	+
DaESP1	-	+	-	-	+
DlESP1	-	+	-	-	+
ItESP1	-	+	-	-	+
SpESP1	-	+	-	-	+
ChNSP1	-	-	+	-	+
ItNSP1	-	-	+	-	+
SpNSP1	-	-	+	-	+

Activity was tested using crude extracts of bacteria expressing specifier protein cDNAs, 1 mM allyl- or 2 mM benzylglucosinolate and 0.005 units myrosinase in 50 mM MES buffer, pH 6.0. The presence of non-isothiocyanate products (organic thiocyante, epithionitrile, simple nitrile) in dichloromethane extracts of the assay mixtures after 30 min incubation is indicated by +, the absence by - (in comparison to assays with extracts of bacteria harbouring the expression vector without cDNA insert, for chromatograms see Additional file 5: Figure S3).

For seven of the eleven Brassicaceae species subjected to the PCR approach, specifier proteins were identified (Table 1). From *Erysimum hieraciifolium*, only an incomplete ORF was obtained that was not included in further analyses. In three species (*Cardamine hirsuta*, *Isatis tinctoria*, *Schouwia purpurea*), specifier proteins of two different types were identified. This confirms that our PCR approach was a suitable method for identification of specifier protein cDNAs. We can, however, not exclude that species from which no PCR product was obtained possess specifier proteins and that additional specifier proteins present in the same species were not detected due to low transcript abundance or deviating nucleotide sequences.

### Phylogenetic analyses

**Figure 2:**
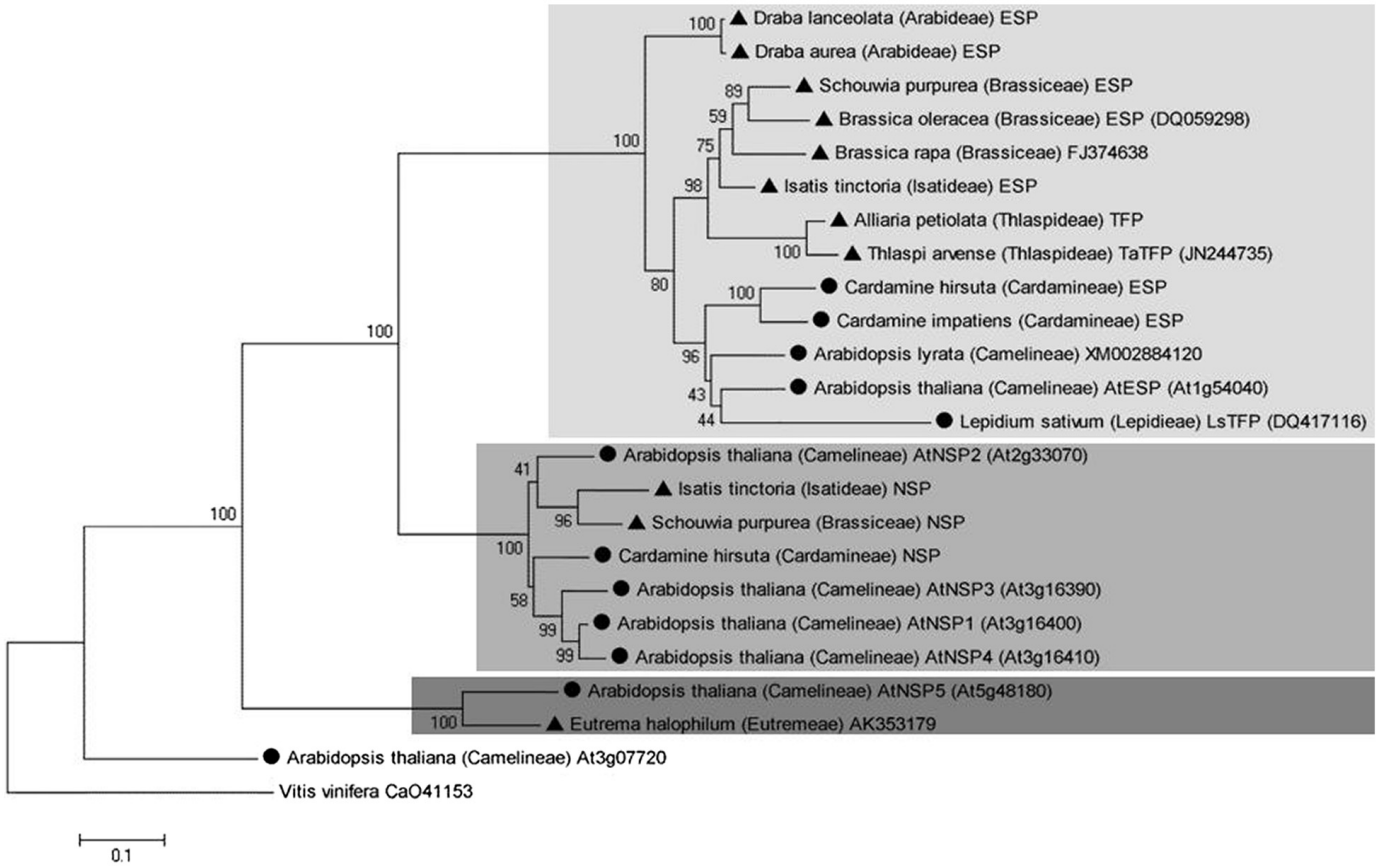
**Phylogenetic tree of specifier protein cDNAs from Brassicaceae. **Full-length nucleotide sequences of 19 biochemically characterized NSPs, ESPs and TFPs (Tab. 1) as well as three putative specifier proteins (Tab. 3) and one homolog of unknown function (At3g07720) were subjected to phylogenetic analysis using the Maximum Likelihood algorithm with 1000 bootstrap repetitions. Bootstrap values are given at the nodes. A homolog from *Vitis vinifera *(Vitaceae) which does not contain glucosinolates was used as an outgroup. Alignment gaps (e.g. JAL domains that are present only in NSPs) are regarded as non-informative posititions in this analysis. Symbols indicate Brassicaceae lineages (lineage I, circle; expanded lineage II, triangle). Boxes indicate clusters (dark grey, AtNSP5 cluster; grey, AtNSP1 cluster; light grey, ESP/TFP cluster). Branch lengths refer to the number of nucleotide substitutions per site. A scale bar is given below the tree.

Together with specifier proteins identified in previous studies, a total of 19 characterized specifier proteins representing nine genera of lineage I and expanded lineage II of the Brassicaceae according to [33] were available for phylogenetic analyses. Additionally, the homolog At3g07720 and three full-length cDNA sequences of putative specifier proteins identified in databases were included (Table 3). Amino acid sequence comparisons showed that amino acid sequence identities are high among all ESP and TFP sequences (59–98%) and between NSP sequences (74–92%) with the exception of AtNSP5 and its homolog from *Eutrema halophilum*, but lower when ESP or TFP sequences are compared with NSP sequences other than AtNSP5 and its homolog (48–63%) (Additional file 6: Table S3). In agreement with this, sequences clustered in three major groups in a phylogenetic tree generated using the Maximum Likelihood algorithm (Figure 2). The first and presumably oldest cluster contained *AtNSP5* and its homolog from *E. halophilum* and was named AtNSP5 cluster. The second cluster was formed by *AtNSP1AtNSP4* and *NSPs* from *Isatis tinctoriaSchouwia purpurea* and *Cardamine hirsuta* and was named AtNSP1 cluster. A third cluster named ESP/TFP cluster contained exclusively *ESP* and *TFP* sequences. Each of the three clusters contained sequences from lineage I and expanded lineage II of the Brassicaceae [33]. In the ESP/TFP cluster, sequences grouped according to the Brassicaceae lineage they were derived from (Figure 2). *At3g07720* with 65% nucleotide identity to *AtNSP5* was located at the basis of the tree. In contrast to *AtNSP1-AtNSP5At3g07720* with unknown function has uncharacterized homologs in a fungal, an algal, and a bryophyte species as well as in higher plant species that do not contain glucosinolates [30]. One of these homologs, *CaO41153* from *Vitis vinifera* (Vitaceae), was used as outgroup and represented the root of the tree. Principally, the same tree topology was obtained when amino acid sequences and other algorithms were used (Additional file 7: Figures S4, Additional file 8: S5, Additional file 9: S6).

**Table 3:** Putative specifier proteins included in phylogenetic analyses

**Database Reference number**	**Source**	**ORF (bp)**	**JAL-domains**	**Kelch-domains**	**Evidence**
FJ374638	*Brassica rapa *subsp. *pekinensis*	1032	-	4	mRNA, complete coding sequence
XM_002884120	*Arabidopsis lyrata*	1023	-	4	genome sequence
AK353179	*Eutrema halophilum *(syn. *Thellungiella halophila*)	975	-	4	mRNA, complete coding sequence[35]

Transcript or genome sequences encoding proteins with highest amino acid sequence identities to specifier proteins are listed with their database reference number. Domain structures are given according to InterProScan predictions.

In four species, pairs of specifier protein paralogs were available. Each pair consisted of one NSP of the AtNSP1 cluster and one ESP. Thus, for each functionally characterized NSP of the AtNSP1-cluster, an ESP paralog could be included in the analysis. Pairwise analyses of the amino acid distances (*d*_aa_) as defined by the number of amino acid substitutions per number of alignment positions corrected for multiple substitutions at the same site and different substitution rates at different alignment positions of these specifier proteins showed higher divergences between NSP and ESP sequences of the same species (paralogs; 0.724-0.940) than within NSP or ESP sequences of different species (orthologs; NSPs 0.153-0.286, ESPs 0.153-0.354) (Table 4; Additional file 10: Table S4). Within *A. thalianad*_aa_ values for comparisons among AtNSP1, AtNSP3 and AtNSP4 were between 0.057 and 0.155 while distances between these proteins and AtNSP2 were 0.194-0.232 (Additional file 10: Table S4). This is in agreement with the known presence of the genes encoding AtNSP1, AtNSP3 and AtNSP4 as tandem genes that likely arose by recent gene duplications. Principally the same result was obtained when nucleotide distances (*d*_na_) as defined by the number of nucleotide substitutions per number of alignment positions corrected for multiple substitutions at the same site and the likelihood of transitions and transversions were determined (Tab. 4, Additional file 10: Table S4). Paralogous *NSP* and *ESP* sequences within one species had higher distances (0.637-0.788; Additional file 10: Table S4) than orthologous *NSP* or orthologous *ESP* sequences (*NSPs* 0.127-0.212, *ESPs* 0.120-0.257%; Additional file 10: Table S4). Together with the topology of the trees, the data suggest one common origin of the genes encoding specifier proteins of the ESP/TFP cluster from genes encoding NSPs. From a biochemical point of view, a monophyletic origin of ESPs/TFPs might be due to aspects of similarity in their catalytic roles. In contrast to simple nitrile formation catalyzed by NSPs, production of epithionitriles and organic thiocyanates requires an intramolecular transfer of a sulfur atom [36-38]. As ESP/TFP sequences cluster according to the Brassicaceae lineages, the gene duplication that led to the split between the AtNSP1 cluster and the ESP/TFP cluster must have happened before the separation of lineages in the core Brassicaceae.

**Table 4:** Amino acid and nucleotide distances of selected NSPs and ESPs

**lineage**		**AtESP**	**ChESP1**	**SpESP1**	**ItESP1**	**AtNSP3**	**ChNSP1**	**SpNSP1**	**ItNSP1**
I	AtESP	-	0.282	0.271	0.266	0.781			
I	ChESP1	0.248	-	0.354	0.299		0.940		
II	SpESP1	0.234	0.256	-	0.153			0.745	
II	ItESP1	0.228	0.257	0.120	-				0.724
I	AtNSP3	0.772				-	0.216	0.201	0.286
I	ChNSP1		0.751			0.155	-	0.153	0.229
II	SpNSP1			0.637		0.181	0.177	-	0.153
II	ItNSP1				0.708	0.212	0.193	0.138	-

Distances are shown for NSPs and ESPs which were identified as paralogous pairs. Note that of the *A. thaliana* NSP sequences, only AtNSP3 is included (see Tab. S4 for all comparisons). Amino acid distances are shown on the right, nucleotide distances on the left as determined by MEGA Vers. 5.05 [39] using the Equal-input + G model (*d*_aa_) and the Kimura-Two-Parameter model + G + I (*d*_na_). Alignment gaps (e.g. JAL domains that are present only in NSPs) are regarded as non-informative posititions in this analysis. The very left column indicates the Brassicaceae lineage according to [33]. Protein abbreviations are as given in Table 1.

Time estimates date the beginning radiation of the core Brassicaceae at 40–50 million years ago, i.e. at about the same time as the *At*α-whole genome duplication is thought to have occurred [33,40]. The *At*α-whole genome duplication is the youngest of three whole genome duplications (α, β, γ) that happened in the evolution of the core Brassicaceae and has first been detected in *A. thaliana* (*At*α). In contrast to the β-whole genome duplication that took place about 70 million years ago in the Brassicales and the γ-whole genome duplication that dates back to early eudicot or angiosperm evolution, the Atα-whole genome duplication is specific for the core Brassicaceae. A possible link between this whole genome duplication and increasing species numbers has been proposed [41,42]. Lineages I and II appeared 36 and 31 million years ago, respectively, or later [33,40], i.e. after the *At*α-whole genome duplication. Consequently, the separation of specifier proteins of the AtNSP1-cluster and the ESP/TFP-cluster dates back to at least about 36 million years ago and might possibly be a consequence of the α-whole genome duplication followed by neofunctionalization. Alternatively, local gene duplication and neofunctionalization may be responsible for the evolution of specifier proteins of the AtNSP1 and the ESP/TFP cluster. Based on this, NSP-ESP/TFP paralogs would be expected in all core Brassicaceae. In four species, NSP-ESP paralogs are functional. In the remaining species analyzed, one of the paralogs may have been lost due to natural selection or it may have escaped our approach.

A closer analysis of the ESP/TFP-cluster revealed that specifier proteins of this cluster form several subgroups. A first branch contained only two ESPs identified from *Draba* species and was separated from all other ESPs/TFPs which clustered according to the Brassicaceae lineages [33] they were identified from. ESPs and TFP of lineage I grouped together in one branch while another branch contained ESPs and TFPs from expanded lineage II. Overall, amino acid distances between ESPs of the same lineage were only slightly lower than amino acid distances between ESPs of different lineages (Table 5, Additional file 10: Table S4). Amino acid distances between TaTFP and ApTFP, respectively, and ESPs from the same lineage (expanded lineage II) were in the same range as those between ESPs. In contrast, amino acid distances were considerably higher between LsTFP and ESPs from the same lineage (lineage I; Table 5, Additional file 10: Table S4). Highest amino acid distances were determined between TFPs of different lineages (Table 5, Additional file 10: Table S4). This suggests that TFPs originated from ESPs after the separation of lineage I and expanded lineage II in the Brassicaceae. Presently, there are no pairs of paralogous ESP and TFP sequences available whose analysis would allow to confirm an independent origin of TFPs from ESPs in the two lineages as suggested by the phylogeny.

**Table 5:** Amino acid and nucleotide distances of ESPs and TFPs

	** *d* **_**aa **_**(%)**	** *d* **_**na **_**(%)**
ESPs lineage I	0.278-0.312	0.213-0.248
ESPs expanded lineage II	0.111-0.294	0.155-0.275
ESPs lineage I - ESPs expanded lineage II	0.123-0.414	0.120-0.319
TFP lineage I - ESPs lineage I	0.479-0.605	0.345-0.408
TFPs expanded lineage II - ESPs expanded lineage II	0.299-0.366	0.232-0.445
TFP lineage I - TFPs expanded lineage II	0.681-0.701	0.531-0.545

Amino acid distances (*d*_aa_) and nucleotide distances (*d*_na_) are given as determined by MEGA Vers. 5.05 using the Equal-input + G model (*d*_aa_) and the Kimura-2-Parameter model + G + I (*d*_na_). Distances between sequences from the same genus are not included. Brassicaceae lineages are according to [33].

**Figure 3:**
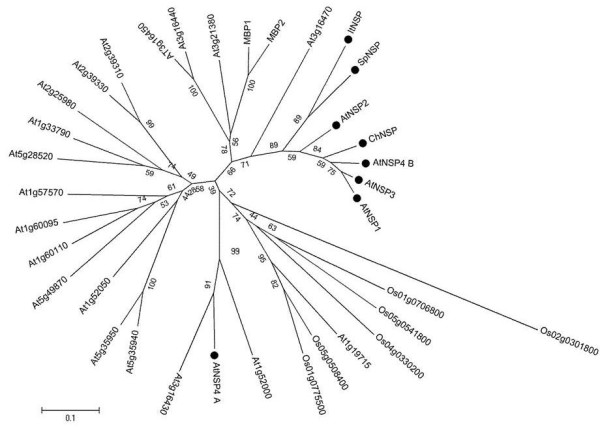
**Phylogenetic tree of JAL domains of NSPs from Brassicaceae as well as homologs composed of JAL domains identified in databases. **Amino acid sequences of JAL domains of eight NSPs (marked with a black circle) and 26 homologs of unknown function from *A. thaliana *or *Oryza sativa* (Poaceae) were subjected to phylogenetic analysis using the Maximum Likelihood algorithm. A homolog from *O. sativa *was used as an outgroup. The two JAL domains of AtNSP4 were considered separately, with the N-terminal domain termed A and the following domain termed B. The tree was generated with 1000 bootstrap repetitions. Bootstrap values are given at the nodes. Branch lengths refer to the number of substitutions per site. A scale bar is given below the tree.

As alignment gaps are considered non-informative in the above analyses, the results represent the phylogenetic relationships based on comparisons among the kelch domain regions of the sequences analyzed. Therefore, we performed a separate analysis of those regions of NSPs that are JAL domains. The two JAL domains of AtNSP4 were used separately with the N-terminal JAL domain termed A and the second JAL domain termed B. The analysis confirmed an earlier study on AtNSP1-AtNSP4 and suggested a monophyletic origin of all JAL domains except JAL domain A of AtNSP4 (Figure 3). Together with the fact that all NSPs of the AtNSP1 cluster and only these NSPs possess a JAL domain of the B-type, this suggests recruitment of this JAL domain early in the evolution of NSPs of the AtNSP1 cluster, likely in conjunction with the gene duplication that gave rise to the split between specifier proteins of the AtNSP1 and the ESP/TFP cluster. JAL domain A is only present in AtNSP4. More data on the architecture of the AtNSP1/ AtNSP3/ AtNSP4 gene cluster in the genome of *A. thaliana* and its relatives are required to find out in which evolutionary time frame JAL domain A was recruited.

### Analysis of selection pressure

Nucleotide substitutions in coding sequences can either be silent (synonymous) or amino acid-changing (non-synonymous). Natural selection mainly acts on non-synonymous substitutions leading to different rates of non-synonymous vs. synonymous substitutions [43]. The ratio of non-synonymous vs. synonymous substitutions can be used to estimate selection pressures acting on a given gene. In comparison of two sequences, the number of non-synonymous substitutions per non-synonymous site (*d*_N_) and the number of synonymous substitutions per synonymous site (*d*_S_) can be determined with correction for multiple substitutions at the same site, different codon frequencies and transversion/transition rates [43]. A non-synonymous vs. synonymous substitution rate ratio ω (ω = *d*_N_ / *d*_S_) <1 indicates purifying (or stabilizing) selection that maintains structural features required for protein function. Equal occurence of non-synonymous and synonymous substitutions over the length of the sequence would result in ω =1 (neutral selection). An ω >1 indicates that positive selection acts on a gene changing the encoded amino acid sequence towards a certain functionality that provides selective advantages.

In order to analyze selection pressures acting on specifier protein genes, we determined non-synonymous vs. synonymous substitution rate ratios (ω) using pairwise comparisons of full-length coding sequences (Additional file 11: Table S5). In no case did we find indications for positive selection. Synonymous substitutions were by far dominating over non-synonymous substitutions over the lengths of the cDNA sequences as indicated by ω < 1. This suggests that purifying selection acts on specifier protein sequences preventing the fixation of mutations that impair functionality. Although ω-values were quite homogenous across all comparisons, lowest ω-values were obtained for NSP-NSP comparisons (0.123-0.435) and NSP-ESP comparisons (0.219-0.351) while ESP-TFP comparisons generally gave higher ω-values (0.344-0.549) and ESP-ESP comparisons showed a broad range of ω-values (0.143-0.627). This may possibly indicate stronger selection pressures acting on NSPs than on the derived ESPs and TFPs and reduced functional constraints associated with the evolution of new biochemical functions. However, ω-values for comparisons within the group of paralogous NSP and ESP sequences were similar for all comparisons (0.25-0.429, Additional file 11: Table S5). Future studies have to show if the ω-values >0.5 detected over the length of the sequences in some of the comparisons are indicative of considerably higher local ω-values to clarify if positive selection has acted on certain sequence motifs. Biochemical characterization of specifier proteins of all three types has shown that TFPs possess additional capabilities (organic thiocyanate formation) as compared to ESPs while ESPs possess additional capabilities (epithionitrile formation) as compared to NSPs [28-30,44]. Thus, the new capabilities do not replace those present in the ancestor but rather add to them. This seems to be true for ESPs that evolved from NSPs following genome or gene duplication as well as for TFPs that evolved from ESPs. In the latter case, there is, at least up to now, no indication for gene duplication events prior to gaining an additional biochemical function.

As pointed out by Kroymann (2011) [2], it is very difficult to dissect the original ecological role of a particular secondary metabolite that has been selected for during evolution. In case of the glucosinolate-myrosinase system, both intact glucosinolates and their breakdown products need to be considered with their effects on a diverse array of herbivores and pathogens, including specialists and generalists [2]. For the evolution of specifier proteins, the glucosinolate profile of a given species must have been an important factor. While NSPs seem to work on glucosinolate aglycones with various side chains [30], the recruitment of ESPs from NSPs can provide selective advantage only in plants that have the biosynthetic capacity to produce aliphatic glucosinolates with a terminal double bond which can be converted to epithionitriles. Such glucosinolates are only found in the relatively young families Brassicaceae and Capparaceae [11,12,40]. Hence, only in those families are ESPs expected to occur. In a similar way, only plants that possess allyl-, 4-methylthiobutyl- or benzylglucosinolate can benefit from the presence of TFPs which form organic thiocyanates during breakdown of only these three glucosinolates due to the ability of their side chains to form stable carbocations [26,36]. Glucosinolates that deliver substrates for NSPs and TFPs are widespread among basal and core Brassicales [11]. However, besides a minor proportion of simple nitrile formed in *T. minor* leaf and flower homogenates, there is no indication for the presence of specifier proteins with a function in glucosinolate breakdown outside the Brassicaceae. To clarify if specifier proteins are an innovation of the Brassicaceae, molecular analyses of more ancient sister families are required.

While the toxicity of isothiocyanates, whose formation does not require specifier proteins, and their role as direct chemical defenses have been proven in various studies [17,23,25], much less is known about the ecological roles of the alternative breakdown products. In several cases, simple nitriles seem to be less toxic than their isothiocyanate counterparts with the same side chain structure [25]. Why does a plant then benefit from investing into the production of specifier proteins if it is well defended by toxic isothiocyanates? A possible explanation is that specifier proteins may help a plant to defend itself against specialist herbivores that evolved adaptations to the glucosinolate-myrosinase system and use glucosinolates and isothiocyanates as cues for host identification. This has been demonstrated experimentally for direct and indirect simple nitrile-mediated plant defense against the cabbage white butterfly (*Pieris rapae*, Lepidoptera: Pieridae) [30,45,46], a representative of the Pierinae which diversified at about the same time as glucosinolate-containing plants [40,47]. Other putative functions of NSPs have been suggested [27]. The fact that NSP and ESP activities are maintained in the same species through natural selection supports different biological roles of simple nitriles and epithionitriles. Interestingly, there is natural variation in *A. thaliana* with respect to expression of functional ESP, and ESP is counterbalanced by modifiers such as *ESM1*[24].

## Conclusions

Phylogenetic analyses of ten newly identified and nine previously known characterized specifier proteins of the Brassicaceae as well as three putative specifier proteins from Brassicaceae found in databases and one homolog of unknown function suggest NSPs to be the ancestral representatives of this group of proteins and provide evidence for a monophyletic origin of ESPs from NSPs. The split between NSPs and ESPs occurred before the diversification of the core Brassicaceae, i.e. at least about 36 million years ago. TFP activity evolved from ESPs later, and phylogenetic analyses indicate that this happened independently in different lineages. Based on the data available to date, specifier proteins might be an innovation of the Brassicaceae that likely expands the defensive repertoire of the Brassicaceae, among others possibly as a response to selection pressures exerted by specialist herbivores that evolved adaptations to the glucosinolate-myrosinase system. The evolution of specifier proteins demonstrates the plasticity of secondary metabolism in the context of an activated plant defense system.

## Methods

### Chemicals and general experimental procedures

Custom-made oligonucleotides were from Invitrogen. Allylglucosinolate (sinigrin) was purchased from AppliChem. Benzylglucosinolate was purified from *Lepidium sativum* L. seeds according to [48]. Myrosinase was purified from *S. alba* seeds [44]. One unit was defined as the amount of myrosinase needed to release 1 μmol glucose per min from 2 mM allylglucosinolate in 50 mM Tris/HCl, pH 7.5, at 37°C as determined with the GOD-PAP kit (Randox laboratories). Protein content was determined with the BCA Protein Assay Kit (Thermo Scientific) according to the manufacturer’s directions using bovine serum albumine as a standard.

### Plant material

Sources of seeds are given in Additional file 12: Table S6. Seeds were germinated in conventional flower soil (Compo sana, COMPO GmbH & Co., KG, Münster, Germany) in a green house at about 22°C and 60–70% relative humidity with a photoperiod of 16 h.

### Glucosinolate analysis

Plant material was frozen in liquid nitrogen, stored at −80°C, and freeze-dried before analysis. Glucosinolate profiles were determined by HPLC of the corresponding desulfoglucosinolates as described previously [49], but without addition of internal standard.

### Glucosinolate hydrolysis products in tissue homogenates

Fresh plant tissue (100–300 mg) was homogenized in 500 μl 50 mM MES buffer, pH 6.0. After 5 min incubation at room temperature, 50 μl internal standard (phenylcyanide, 100 ng μl^-1^ in MeOH) were added, tissue remainings were removed by centrifugation, and the supernatant was extracted twice with 750 μl CH_2_Cl_2_. Samples were concentrated in an air stream to about 200 μl and analyzed by GC-MS as described [29].

### cDNA preparation and degenerate oligonucleotide PCR

Leaves were frozen in liquid nitrogen and stored at −80°C until used for cDNA generation. Total RNA was extracted using ribozol-OLS (OLS OMNI Life Science) or innuPREP Plant RNA Kit (Analytik Jena) according to the manufacturer's instructions and used for reverse transcription with SuperScript III Reverse Transcriptase (Invitrogen) and oligo(dT)_12–18_ primers (Invitrogen) according to the instructions of the manufacturer. Based on an amino acid sequence alignment of known specifier proteins, conserved regions within NSP sequences and within ESP/TFP sequences were identified (Additional file 3: Figure S2). Degenerate primers P1, P2, P5, and P6 (Additional file 4: Table S2) were designed based on the nucleotide sequences in these regions and used at 0.2 μM in PCR reactions containing 1.6 μl cDNA preparation, 0.1 mM of each dNTP, 2 mM MgCl_2_, and 0.5 μl peqGOLD *Taq* DNA polymerase (PEQLAB) in a total volume of 50 μl peqGOLD *Taq* buffer. The temperature program (Biometra Professional Basic thermocycler) was as follows: 94°C for 3 min, followed by 35 cycles of 94°C for 75 s, 54-58°C (depending on primer combination) for 150 s, and 72°C for 1 min, and a final incubation at 72°C for 10 min. PCR products were cloned into pGEM-T Easy (Promega) according to the manufacturer’s protocol and sequenced (GATC Biotech AG, Konstanz, Germany).

### RACE-PCR

Gene-specific primers (Additional file 4: Table S2) were designed based on the cDNA fragments obtained by PCR with degenerate primers. For 3'-RACE, first strand cDNA was generated from 1 μg total RNA by SuperScript III Reverse Transcriptase (Invitrogen) using oligo(dT)-anchor primer P7 (Additional file 4: Table S2) according to the instructions of the manufacturer. Of this reaction, 1 μl was subjected to PCR with a gene-specific sense primer and primer P7 (each 0.2 μM) using peqGOLD *Taq* DNA polymerase (PEQLAB) or *Pfu* DNA polymerase (Fermentas) in the appropriate buffer (with Mg^2+^) containing 0.1 mM of each dNTP. The temperature program (Biometra Professional Basic thermocycler) was as follows: 94°C for 3 min, followed by 35 cycles of 94°C for 75 s, appropriate annealing temperature for 150 s, and 72°C for 1 min, and a final incubation at 72°C for 10 min. If required, this was followed by another PCR with a downstream gene-specific sense primer and primer P7 (nested 3'PCR; Additional file 4: Table S2). For 5'-RACE, first strand cDNA was generated from 1 μg total RNA by SuperScript III Reverse Transcriptase (Invitrogen) using a gene-specific antisense primer according to the instructions of the manufacturer. The cDNA (10 μl of the reaction) was polyadenylated by Terminal Desoxynucleotidyl Transferase (Fermentas) in a total volume of 25 μl according to the manufacturers protocol. Of this reaction, 1 μl was subjected to PCR with oligo(dT)-anchor primer (P7) and a gene-specific antisense primer located upstream of the first gene-specific primer (each 0.2 μM; Additional file 4: Table S2). PCR conditions and temperature program were as described for 3'-RACE-PCR. If required, this was followed by another PCR with another upstream gene-specific antisense primer and primer P7 (nested 5'PCR; Additional file 4: Table S2)). PCR products were cloned into pGEM-T Easy (Promega) according to the manufacturer’s protocol and sequenced (GATC Biotech AG, Konstanz, Germany; Eurofins MWG GmbH, Ebersberg, Germany).

### Cloning of full-length cDNAs and heterologous expression

Sequence information of the fragments obtained by RACE-PCR was used to design gene-specific sense and antisense primers for amplification of the full-length cDNAs or ORFs, respectively (Additional file 4: Table S2). PCR reactions were set up in a total volume of 50 μl *Pfu* buffer with MgSO_4_ (Fermentas) containing 0.2 mM of each dNTP, 0.2 μM of each primer, 1 μl cDNA preparation, and 0.5 μl *Pfu* DNA polymerase (Fermentas). The temperature program (Biometra Professional Basic thermocycler) was as follows: 95°C for 2 min, followed by 35 cycles of 95°C for 1 min, appropriate annealing temperature for 1 min, and 72.5°C for 1 min, and a final incubation at 72.5°C for 10 min. PCR products were cloned and sequenced (GATC Biotech AG, Konstanz, Germany; Eurofins MWG GmbH, Ebersberg, Germany). The sequences were confirmed by independent amplification of the ORFs from 1 μl cDNA using 0.2 μM of each gene-specific primer equipped with USER-compatible ends [50], 0.2 mM of each dNTP, and 1.25 units *Pfu* Turbo Cx Hotstart DNA polymerase (Stratagene) in a total volume of 25 μl *Pfu* buffer (Stratagene). The temperature program was as follows: 95°C for 3 min, 35 cycles of 95°C for 45 s, 58°C for 1 min, and 72°C for 2 min, and a final incubation at 72°C for 10 min. The products of this reaction were cloned into modified pET52(b) + vectors (Novagen). In these modified vectors, a USER cassette [50] either including or not including the coding sequence for an N-terminal Strep-Tag II and an HRV 3 C cleavage site had been introduced between the *Nco*I restriction site and the Strep-Tag II coding sequence of the original pET52(b) + vector. Sequences were verified by sequencing (GATC Biotech AG, Konstanz, Germany; Eurofins MWG GmbH, Ebersberg, Germany), and the constructs were used for heterologous expression according to [29]. Crude bacterial extracts were used for specifier protein assays.

### Specifier protein assays

Assays were carried out in a total volume of 500 μl 50 mM MES-buffer, pH 6.0, containing 1 mM benzylglucosinolate or 2 mM allylglucosinolate and crude bacterial extract corresponding to 1.5 mg total protein and were started by addition of 0.005 units of myrosinase. After 30 min incubation at room temperature, 50 μl of phenylcyanide (100 ng μl^-1^ in MeOH) were added as internal standard and the mixture was extracted twice with 750 μl CH_2_Cl_2_. Organic phases were pooled, dried over Na_2_SO_4_, concentrated under an air stream to about 200 μl and analyzed by GC-MS as described [29].

### Data base search

Blastn was used to search the nucleotide data base at NCBI using cDNA sequences of known specifier proteins as queries. Hits were included in the phylogenetic analyses if they represented complete ORFs with an identity of at least 60% with those of characterized specifier proteins. Using *BoESP* as a query, two nucleotide sequences met these criteria (FJ374638, XM002884). Using AtNSP5 as a query, one sequence met these criteria (AK353179).

### Sequence and phylogenetic analyses

Motif and pattern searches were done with InterProScan at http://www.ebi.ac.uk/Tools/pfa/iprscan/). Multiple sequence alignments and pairwise sequence comparisons were performed with the ClustalW option implemented in MEGA 5.05 [39]. To determine amino acid and nucleotide sequence identities, each sequence pair was aligned and analyzed separately. MEGA 5.05 [39] was used for the construction of phylogenetic trees and for the calculation of amino acid and nucleotide distances as well as non-synonymous and synonymous substitution rates. Alignments of nucleotide and amino acid sequences were edited manually according to the Pam250-matrix prior to distance calculations. For calculation of *d*_na_, we corrected for multiple nucleotide substitutions at the same site. Corrections were based on the Kimura-2-parameter model taking invariable positions (+I) and varying substitution rates among sites (+G) into account. For calculation of *d*_aa_, corrections were implemented for varying substitution rates among sites (+G) based on the Equal input model. Phylogenetic trees of nucleotide sequences were based on the Kimura-2-parameter model, the WAG model corrected for varying substitution rates among sites (+G) was used for phylogenetic trees of amino acid sequences. For the construction of phylogenetic trees by the Neighbor Joining Method and the Maximum Likelihood Method, alignment gaps were disregarded when more than 95% of the sequences were non-informative in this position. Close-Neighbor-Interchange algorithm was applied for construction of trees by the Maximum Likelihood and the Maximum Parsimony Method. Analyses were performed with 1000 bootstrap repetitions. Non-synonymous and synonymous substitution rates were calculated based on the Kimura-2-parameter model.

## Abbreviations

NSP: Nitrile-specifier protein; ESP: Epithiospecifier protein; TFP: Thiocyanate-forming protein; JAL: Jacalin-related lectin.

## Competing interests

The authors declare that they have no financial and no other competing interests.

## Authors’ contributions

JCK performed phytochemical analyses, cloned and characterized specifier proteins, and carried out phylogenetic analyses. MB performed phytochemical analyses. UW designed the study together with JCK and MB and analyzed data. UW wrote the manuscript with comments from all authors. All authors read and approved the final manuscript.

## Supplementary Material

Additional file 1 **Table S1. **Glucosinolate classes and hydrolysis product types in species of the Brassicales. The plant organs indicated were analyzed for glucosinolates by HPLC after derivatization to desulfoglucosinolates. For the analysis of glucosinolate hydrolysis products, fresh plant material was homogenized in aqueous solution, and dichloromethane extracts of the homogenates were analyzed by GC-MS. Glucosinolates were assigned to structural classes according to the structure of their side chains as follows: **A1**, aliphatic: methylthio-/methylsulfinyl-; **A2**, aliphatic: terminal double bond; **A3**, aliphatic: terminal double bond and 2-hydroxy-group; **A4**, aliphatic: other than A1-A3; **B1**, benzyl-; **B2**, 4-hydroxybenzyl-; **B3**, 2-hydroxy-2-phenylethyl-; **B4**, Phe- and Tyr-derived other than B1-B3; **C**, indolic. Glucosinolate hydrolysis product types are abbreviated as follows: **I**, isothiocyanate; **N**, simple nitrile; **EN**, epithionitrile; **OX**, oxazolidine-2-thione; **T**, organic thiocyanate. An X indicates the identification of at least one representative of a glucosinolate class or a hydrolysis product type in the given plant material (n.i., not investigated). Most species were of the Brassicaceae. For species of other families, the family is given below the species name. Click here for file

Additional file 2 **Figure S1. **Glucosinolate hydrolysis products in *Tropaeolum minus* . Fresh leaves (**A**) or flowers (**B**) were homogenized in aqueous buffer, and dichloromethane extracts of the homogenates were analyzed by GC-MS. Representative chromatograms (total ion current) are shown. **1**, benzylisothiocyanate; **2**, phenylacetonitrile; **IS**, internal standard (phenylcyanide). Chromatograms in A and B were recorded with three years difference explaining the changed retention times. Click here for file

Additional file 3 **Figure S2. **Alignment of previously known specifier proteins for primer design. Protein names are as given in Table 1. Amino acid sequences were aligned with the ClustalW option implemented in MEGA 5.05 [39]. White letters on black background indicate amino acid residues that are identical in at least 80% of the sequences, white letters on gray background indicate amino acid residues that are similar in at least 80% of the sequences. In case of AtNSP1 and AtNSP2, partial amino acid sequences starting with amino acid 120 and 121, respectively, are shown. For all other proteins, full-length amino acid sequences are included. Green boxes indicate regions of high amino acid identity that were used to design degenerate primers P1, P2, P5, and P6 (Table S2). Click here for file

Additional file 4 **Table S2. **Oligonucleotide primers for cDNA isolation. Sequences are given starting with the 5' end. Mixed nucleotide codes are: D, A + G + T; K, G + T; M, A + C; R, A + G; S, C + G; W, A + T; Y, C + T. Protein names are as given Table 1. Click here for file

Additional file 5 **Figure S3. **Assignment of newly identified specifier proteins to the ESP, TFP or NSP type. Activity was tested using crude extracts of bacteria expressing specifier protein cDNAs, 1 mM allylglucosinolate (**A-K**) or 2 mM benzylglucosinolate (**L-V**) and 0.005 units myrosinase in 50 mM MES buffer, pH 6.0. Shown are total ion current traces of GC-MS chromatograms recorded of dichloromethane extracts of the assay mixtures after 30 min incubation. Products of allylglucosinolate breakdown are simple nitrile (1), organic thiocyanate (2), isothiocyanate (3), and epithionitrile (4). Products of benzylglucosinolate are simple nitrile (5), and isothiocyanate (6). (X) indicates the peak of the internal standard. The following cDNAs were used: **A, L**, no cDNA (empty vector control); **B, M**, *ApTFP*; **C, N**, *ChESP*; **D, O**, *CiESP*; **E, P**, *DaESP*; **F, Q**, *DlESP*; **G, R**, *ItESP*; **H, S**, *SpESP*; **I, T**, *ChNSP*; **J, U**, *ItNSP*; **K, V**, *SpNSP*. Click here for file

Additional file 6 **Table S3. **Amino acid and nucleotide sequence identities of specifier proteins/ specifier protein cDNAs. Amino acid sequence identities are shown on the right, nucleotide sequence identities on the left as determined in separate comparisons of each sequence pair by ClustalW implemented in MEGA Vers. 5.05. Click here for file

Additional file 7 **Figure S4. **Phylogenetic tree of specifier proteins from Brassicaceae. Full-length amino acid sequences of 19 biochemically characterized NSPs, ESPs and TFPs (Table 1) as well as three putative specifier proteins (Table 3) and one homolog of unknown function (At3g07720) were subjected to phylogenetic analysis using the Maximum Likelihood algorithm with 1000 bootstrap repetitions. Click here for file

Additional file 8 **Figure S5. **Neighbor joining tree of specifier protein cDNAs from Brassicaceae. Full-length nucleotide sequences of 19 biochemically characterized NSPs, ESPs and TFPs (Table 1) as well as three putative specifier proteins (Table 3) and one homolog of unknown function (At3g07720) were subjected to phylogenetic analysis using the Neighbor joining algorithm with 1000 bootstrap repetitions. Bootstrap values are given at the nodes. A homolog from *Vitis vinifera* (Vitaceae) which does not contain glucosinolates was used as an outgroup. Alignment gaps (e.g. JAL domains that are present only in NSPs) are regarded as non-informative posititions in this analysis. Branch lengths refer to the number of substitutions per site. A scale bar is given below the tree. Click here for file

Additional file 9 **Figure S6. **Maximum Parsimony tree of specifier protein cDNAs from Brassicaceae. Full-length nucleotide sequences of 19 biochemically characterized NSPs, ESPs and TFPs (Table 1) as well as three putative specifier proteins (Table 3) and one homolog of unknown function (At3g07720) were subjected to phylogenetic analysis using the Maximum Parsimony algorithm with 1000 bootstrap repetitions. Bootstrap values are given at the nodes. A homolog from *Vitis vinifera *(Vitaceae) which does not contain glucosinolates was used as an outgroup. Alignment gaps (e.g. JAL domains that are present only in NSPs) are regarded as non-informative posititions in this analysis. Click here for file

Additional file 10 **Table S4. **Amino acid and nucleotide distances of specifier proteins/ specifier protein cDNAs. Amino acid distances are shown on the right, nucleotide distances on the left as determined by MEGA Vers. 5.05 using the Equal-input + G model (*d*_aa_) and the Kimura-Two-Parameter model + G + I (*d*_na_). Alignment gaps (e.g. JAL domains that are present only in NSPs) are regarded as non-informative posititions in this analysis. Click here for file

Additional file 11 **Table S5. **Non-synonymous vs. synonymous substitution rate ratios of specifier protein cDNAs.*d*_N_ and *d*_S_ were calculated by MEGA 5.05 from pairwise comparisons based on the Kimura-2-parameter model and used to determine ω as the *d*_N_ / *d*_S_ ratio. Click here for file

Additional file 12 **Table S6. **Sources of seeds for growing plants for phytochemical analysis and cDNA isolation. Click here for file
