# Supplementary material for: Evolution of specifier proteins in glucosinolate-containing plants
Source: BMC Evol Biol. 2012 Jul 28;12:127. doi: 10.1186/1471-2148-12-127 (PMC3482593; doi:10.1186/1471-2148-12-127)
Supplement: Additional file 1 — Table S1. Glucosinolate classes and hydrolysis product types in species of the Brassicales. The plant organs indicated were analyzed for glucosinolates by HPLC after derivatization to desulfoglucosinolates. For the analysis of glucosinolate hydrolysis products, fresh plant material was homogenized in aqueous solution, and dichloromethane extracts of the homogenates were analyzed by GC-MS. Glucosinolates were assigned to structural classes according to the structure of their side chains as follows: A1, aliphatic: methylthio-/methylsulfinyl-; A2, aliphatic: terminal double bond; A3, aliphatic: terminal double bond and 2-hydroxy-group; A4, aliphatic: other than A1-A3; B1, benzyl-; B2, 4-hydroxybenzyl-; B3, 2-hydroxy-2-phenylethyl-; B4, Phe- and Tyr-derived other than B1-B3; C, indolic. Glucosinolate hydrolysis product types are abbreviated as follows: I, isothiocyanate; N, simple nitrile; EN, epithionitrile; OX, oxazolidine-2-thione; T, organic thiocyanate. An X indicates the identification of at least one representative of a glucosinolate class or a hydrolysis product type in the given plant material (n.i., not investigated). Most species were of the Brassicaceae. For species of other families, the family is given below the species name. [file 1471-2148-12-127-S1.pdf]

Tab. S1: Glucosinolate classes and hydrolysis product types in species of the Brassicales. The plant organs indicated were analyzed for glucosinolates by HPLC after derivatization to desulfoglucosinolates. For the analysis of glucosinolate hydrolysis products, fresh plant material was homogenized in aqueous solution, and dichloromethane extracts of the homogenates were analyzed by GC-MS. Glucosinolates were assigned to structural classes according to the structure of their side chains as follows: **A1**, aliphatic: methylthio-/methylsulfinyl-; **A2**, aliphatic: terminal double bond; **A3**, aliphatic: terminal double bond and 2-hydroxy-group; **A4**, aliphatic: other than A1-A3; **B1**, benzyl-; **B2**, 4-hydroxybenzyl-; **B3**, 2-hydroxy-2-phenylethyl-; **B4**, Phe- and Tyr-derived other than B1-B3; **C**, indolic. Glucosinolate hydrolysis product types are abbreviated as follows: **I**, isothiocyanate; **N**, simple nitrile; **EN**, epithionitrile; **OX**, oxazolidine-2-thione; **T**, organic thiocyanate. An X indicates the identification of at least one representative of a glucosinolate class or a hydrolysis product type in the given plant material (n.i., not investigated). Most species were of the Brassicaceae. For species of other families, the family is given below the species name.

**Species**	**Organ**	**Structural classes of glucosinolates**									**Types of glucosinolate hydrolysis products**				
		**A1**	**A2**	**A3**	**A4**	**B1**	**B2**	**B3**	**B4**	**C**	**I**	**N**	**EN**	**OX**	**T**
Alliaria petiolata	seedlings		X			X				X	X	X	X		X
Alyssum alpestre	leaves	X								X	X	X			
	roots	X								X	X				
	seeds	X									X				
Arabis glabra	leaves	X								X	X				
	roots	X								X					
	seeds	X													
Barbarea intermedia	leaves					X			X	X				X	
	roots							X	X	X	n.i.	n.i.	n.i.	n.i.	n.i.
	seeds							X	X	X	X			X	
Barbarea stricta	leaves						X	X		X				X	
	roots							X	X	X	X			X	
	seeds						X	X		X				X	
Barbarea vulgaris	seeds							X	X	X	X			X	
Camelina microcarpa	roots	X													
	flowers	X													
	siliques	X								X					
	seeds	X													
Capsella bursa-pastoris	roots	X													
	flowers		X								X				
	seeds	X	X												

Tab. S1, continued

Species	Organ	A1	A2	A3	A4	B1	B2	B3	B4	C	I	N	EN	OX	T
Capsella rubella	leaves	X													
	seedlings	X													
	flowers										X				
	seeds	X									n.i.	n.i.	n.i.	n.i.	n.i.
Cardamine hirsuta	leaves	X	X			X	X		X	X	X	X	X		
	roots		X			X			X	X	X	X	X		
	seeds		X			X				X	X	X	X		
	siliques		X			X				X	X	X	X		
Cardamine impatiens	leaves		X	X		X				X		X	X		
	seeds		X	X							X		X		
Cleome hassleriana	leaves				X	X				X	X				
(Cleomaceae)	roots				X					X	X				
	seeds			X	X	X					X			X	
Cleome spinosa	leaves				X					X					
(Cleomaceae)	seeds		X		X					X	n.i.	n.i.	n.i.	n.i.	n.i.
	flowers				X					X	X				
Diplotaxis muralis	leaves	X	X		X		X			X	X				
	seeds	X									X	X			
	flowers	X	X		X					X	X				
	roots	X					X			X	X	X			
Draba aurea	leaves	X	X						X	X			X		
	roots	X	X							X					
	seeds	n.i.	n.i.	n.i.	n.i.	n.i.	n.i.	n.i.	n.i.	n.i.	X		X		
Draba lanceolata	leaves	X	X	X									X		
	roots	X	X	X											
	seeds	n.i.	n.i.	n.i.	n.i.	n.i.	n.i.	n.i.	n.i.	n.i.	X	X	X	X	
Draba muralis	leaves								X		X	X			
	roots						X		X	X					
	seeds						X		X	X	X				
Eruca sativa	leaves	X	X		X					X	X				
	seedlings	X			X						n.i.	n.i.	n.i.	n.i.	n.i.
	seeds	X									X				
	flowers	X									X				
	siliques	X			X						n.i.	n.i.	n.i.	n.i.	n.i.
	flower buds	X			X						n.i.	n.i.	n.i.	n.i.	n.i.
	roots	n.i.	n.i.	n.i.	n.i.	n.i.	n.i.	n.i.	n.i.	n.i.	X				

Tab. S1, continued

**Species**	**Organ**	**A1**	**A2**	**A3**	**A4**	**B1**	**B2**	**B3**	**B4**	**C**	**I**	**N**	**EN**	**OX**	**T**
Erysimum cheiri	leaves	X								X	X				
	seeds	X			X						X				
	roots	X								X	X				
Erysimum hieraciifolium	leaves	X	X							X	X				
	seeds	X	X		X					X	X				
	roots	X	X							X	X	X			
Iberis amara	leaves	X								X	X				
	seeds	X									X				
	flowers	n.i.	n.i.	n.i.	n.i.	n.i.	n.i.	n.i.	n.i.	n.i.	X	X			
	roots	X								X	X				
Isatis tinctoria	leaves	n.i.	n.i.	n.i.	n.i.	n.i.	n.i.	n.i.	n.i.	n.i.		X			
	seedlings		X	X						X	n.i.	n.i.	n.i.	n.i.	n.i.
	seeds		X	X						X					
	roots	n.i.	n.i.	n.i.	n.i.	n.i.	n.i.	n.i.	n.i.	n.i.		X			
Limnanthes douglasii	leaves								X	X	X				
(Limnanthaceae)	seeds								X		X				
	roots								X		X				
Moringa oleifera	leaves		X			X	X				X				
(Moringaceae)	seeds						X		X		n.i.	n.i.	n.i.	n.i.	n.i.
Reseda lutea	leaves					X				X					
(Resedaceae)	seeds					X	X			X					
	roots					X	X		X	X	X				
Reseda luteola	leaves							X	X	X				X	
(Resedaceae)	seeds	n.i.	n.i.	n.i.	n.i.	n.i.	n.i.	n.i.	n.i.	n.i.				X	
	roots							X	X	X	X			X	
Schouwia purpurea	leaves	n.i.	n.i.	n.i.	n.i.	n.i.	n.i.	n.i.	n.i.	n.i.		X	X		
	seedlings		X	X	X					X		X	X		
Tropaeolum minus	leaves	n.i.	n.i.	n.i.	n.i.	n.i.	n.i.	n.i.	n.i.	n.i.	X	X			
(Tropaeolaceae)	seeds					X	X		X		n.i.	n.i.	n.i.	n.i.	n.i.
	flowers					X	X				X	X			
