# Supplementary material for: Evolution of specifier proteins in glucosinolate-containing plants
Source: BMC Evol Biol. 2012 Jul 28;12:127. doi: 10.1186/1471-2148-12-127 (PMC3482593; doi:10.1186/1471-2148-12-127)
Supplement: Additional file 2 — Figure S1. Glucosinolate hydrolysis products in Tropaeolum minus . Fresh leaves (A) or flowers (B) were homogenized in aqueous buffer, and dichloromethane extracts of the homogenates were analyzed by GC-MS. Representative chromatograms (total ion current) are shown. 1, benzylisothiocyanate; 2, phenylacetonitrile; IS, internal standard (phenylcyanide). Chromatograms in A and B were recorded with three years difference explaining the changed retention times. [file 1471-2148-12-127-S2.pdf]

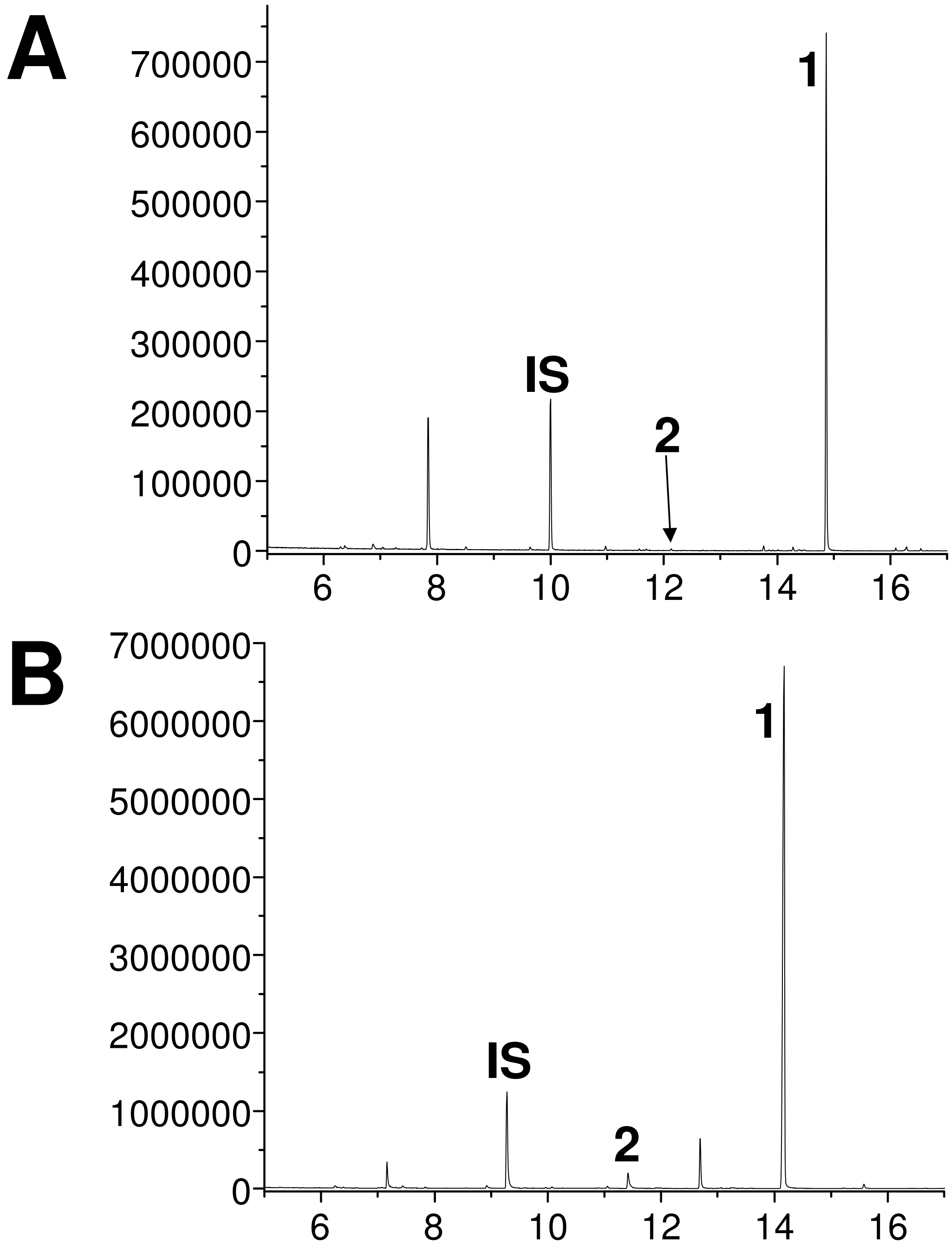

Fig. S1: Glucosinolate hydrolysis products in *Tropaeolum minus*. Fresh leaves (**A**) or flowers (**B**) were homogenized in aqueous buffer, and dichloromethane extracts of the homogenates were analyzed by GC-MS. Representative chromatograms (total ion current) are shown. **1**, benzylisothiocyanate; **2**, phenylacetonitrile; **IS**, internal standard (phenylcyanide). Chromatograms in A and B were recorded with three years difference explaining the changed retention times.
