# Supplementary material for: Evolution of specifier proteins in glucosinolate-containing plants
Source: BMC Evol Biol. 2012 Jul 28;12:127. doi: 10.1186/1471-2148-12-127 (PMC3482593; doi:10.1186/1471-2148-12-127)
Supplement: Additional file 3 — Figure S2. Alignment of previously known specifier proteins for primer design. Protein names are as given in Table 1. Amino acid sequences were aligned with the ClustalW option implemented in MEGA 5.05 [39]. White letters on black background indicate amino acid residues that are identical in at least 80% of the sequences, white letters on gray background indicate amino acid residues that are similar in at least 80% of the sequences. In case of AtNSP1 and AtNSP2, partial amino acid sequences starting with amino acid 120 and 121, respectively, are shown. For all other proteins, full-length amino acid sequences are included. Green boxes indicate regions of high amino acid identity that were used to design degenerate primers P1, P2, P5, and P6 (Table S2). [file 1471-2148-12-127-S3.pdf]

```
AtNSP1     1 MAQKLEAKGGEMGDVWDDGVYENVRKVYVGQAQYGIAFVKFEYVNGSQVVVGDEHGKKTE
AtNSP2     1 MVQKVEARGGEIGDVWDDGAYDGVRKVYVGQGEDGIAFVKFEYVNGSQEVVGDERGKKTL
BoESP      1 ------------------------------------------------------------
AtESP      1 ------------------------------------------------------------
LsTFP      1 ------------------------------------------------------------
AtNSP5     1 ------------------------------------------------------------

AtNSP1    61 LGVEEFEIDADDYIVYVEGYREKVNDMTS-EMITFLSIKTFKGKTSHPIEKRPGVKFVLH
AtNSP2    61 LGAEEFEVDPDDYIVYVEGYHEKVFGVTTKEIISTLTFKTYKGKTSPPFGIVSGTKFVLQ
BoESP      1 ------------------------------------------------------------
AtESP      1 ------------------------------------------------------------
LsTFP      1 ------------------------------------------------------------
AtNSP5     1 ------------------------------------------------------------

AtNSP1   120 GGKIVGFHGRSTDVLHSLGAYVSLSSTIKILGKWIKVEQKG-EGPGLRCSHGIAQVGNKI
AtNSP2   121 GGKIVGFHGRSTDVLHSLGAYISSPATPKIRGKWIKVEQKG-EGPGPRCSHDIAQVGNKI
BoESP      1 -------------------------MAPSVQGEWIKVEQKGGQTPGPRSSHGIAVVGDKL
AtESP      1 -------------------------MAPTLQGQWIKVGQKGGTGPGPRSSHGIAAVGDKL
LsTFP      1 -------------------------MALTLQGEWIKIEQKGGPAPGPRSSHCMAVVGDKL
AtNSP5     1 --------------------------MCPVENKWLKVGQKG-AGPGARSSHAMTVVGNKV
                       P1                                     P5
AtNSP1   179 YSFGGEFTPNQPIDKHLYVFDLETRTWSISPATGDVPHLSCLGVRMVSVGSTLYVFGGRD
AtNSP2   180 FSFGGELTPNQPIDKHLYVFDLETRTWSISPATGDVPNLSCLGVRMVSIGSSLYVFGGRD
BoESP     36 YSFGGELTPNISIDKDLYVFDFNTHTWSISPSKGVAPDVKALGTRMVSVGTKLYLFGGRD
AtESP     36 YSFGGELTPNKHIDKDLYVFDFNTQTWSIAQPKGDAPTVSCLGVRMVAVGTKIYIFGGRD
LsTFP     36 YMFGGELKPQFHLDKHLYVFDFKTNTWSIAEPKGEAPSLSCLGVRMVAVGTKIYIFGGRD
AtNSP5    34 YCFGGELKPTIHIDNDLYVFDLETQEWSIAPATGEAP-FPCFGVSMVTIGSTIYVYGGRD

AtNSP1   239 ASRQYNGFYSFDTTTNEWKLLTPVEE--GPTPRSFHSMAADEENVYVFGGVS------AT
AtNSP2   240 ASRKYNGFYSFDTTKNEWKLLTPVEQ--GPTPRSFHSMTADENNVYVFGGVS------AT
BoESP     96 ENKKFDDFYSYDTVTNEWTKLTILDQEGGPEARTYHSMASDENHVYVFGGVSKGGTNKTP
AtESP     96 ENRNFENFRSYDTVTSEWTFLTKLDEVGGPEARTFHSMASDENHVYVFGGVSKGGTMNTP
LsTFP     96 ENRNYSDFYSYDTVKKEWKFLTKLDEERVPEARSFPAIAADDNHVYIFGGVSKGGVQSTP
AtNSP5    93 DKRRYNGLHSYDTETNEWKLLAPVEE--GLPGRSYHSMAGDDRKVYVFGGVT------AK

AtNSP1   291 ARLNTLDSYNIVDKKWFHCSTPGD---SLTARGGAGLEVVQGKVWVVY---------GFN
AtNSP2   292 VRLKTLDAYNIVDHKWVQCSTPGG---SCSVRGGAGLEVVQGKVWVVY---------GFN
BoESP    156 FRFRTIEAYNIADGKWSQLPDPGEQFPRFERRGGAGFVVVQGKIWVVYGFATSPDPNGKN
AtESP    156 TRFRTIEAYNIADGKWAQLPDPGD---NFEKRGGAGFAVVQGKIWVVYGFATSIVPGGKD
LsTFP    156 FRFKSTIVYNIAEGTWSQLPNPGP---DFEPRGGAGLAVIDKKLWVVCGFANS-TSGGIN
AtNSP5   145 GRVNTLHAYDVVDQKWVEYPAAGE---ACKGRGAPGLVVVEGRIWVLF---------GFD

AtNSP1   339 GCEVDDVHYYDPVQDKWTQVETFGVRPSERSVFASAAIGKHIVIFGGEIAMDPLAHVGPG
AtNSP2   340 GCEVDDVHCYDPAQDKWTQVETFGEKPCARSVFASAVVGKHILVFGGEIAMDPKAHEGPG
BoESP    216 DYESDQVQFYDPATQKWTEVETKGDKPSARSVFGHAVVGKYILIFGGETWPDPKAHLGPG
AtESP    213 DYESNAVQFYDPASKKWTEVETTGAKPSARSVFAHAVVGKYIIIFAGEVWPDLNGHYGPG
LsTFP    212 DYNSNKVQYYDLVSGKWIEVKTSGVKPSGRSVFAYAVIGKQIVIYGGEIFRDENGHLGPG
AtNSP5   193 GNELGDIHCFDLASEQWKAVETTGDVPAARSVFPAVSYGKYIVIYGGEEEPHELMHMGAG
              P2                                         P6
AtNSP1   399 QLTDGTFALDTETLQWERLDKFGGEEETPSSRGWTASTTATIDGKKGLVMHGGKAPTNDR
AtNSP2   400 QLSGGTFALDTETLKWEKLDKLGEEEETPSIRGWSASTTGTIDGKKGLVMFGGKAQTNDR
BoESP    276 TLSDEGFALDTETLVWERFGG-GAEPGQ---LGWPGYTTATVYGKKGLLMHGGKRPTNNR
AtESP    273 TLSNEGYALDTETLVWEKLGEEGAPAIP---RGWTAYTAATVDGKNGLLMHGGKLPTNER
LsTFP    272 TMSNEGYALDTETLVWEKLVDGGEPMTP---LGWTANCTGTVYGKTGLLMHGGKQPFNNR
AtNSP5   253 KMSGEVYQLDTETLVWERIVCGNEEEKP-SQRGWCAFTKAVKDGEEGLLVHGGNSPTNER

AtNSP1   459 FDDLFFYGIDSA---
AtNSP2   460 FGDLFFYGVDSA---
BoESP    332 TDELYFYAVNSA---
AtESP    330 TDDLYFYAVNSA---
LsTFP    329 TDDFYFYSF------
AtNSP5   312 LDDLVFWGFSHLNVN
```

Fig. S2: Alignment of previously known specifier proteins for primer design. Protein names are as given in Tab. 1. Amino acid sequences were aligned with the ClustalW option implemented in MEGA 5.05 [49]. White letters on black background indicate amino acid residues that are identical in at least 80 % of the sequences, white letters on gray background indicate amino acid residues that are similar in at least 80 % of the sequences. In case of AtNSP1 and AtNSP2, partial amino acid sequences starting with amino acid 120 and 121, respectively, are shown. For all other proteins, full-length amino acid sequences are included. Green boxes indicate regions of high amino acid identity that were used to design degenerate primers P1, P2, P5, and P6 (Table S2).
