# Supplementary material for: Evolution of specifier proteins in glucosinolate-containing plants
Source: BMC Evol Biol. 2012 Jul 28;12:127. doi: 10.1186/1471-2148-12-127 (PMC3482593; doi:10.1186/1471-2148-12-127)
Supplement: Additional file 4 — Table S2. Oligonucleotide primers for cDNA isolation. Sequences are given starting with the 5' end. Mixed nucleotide codes are: D, A + G + T; K, G + T; M, A + C; R, A + G; S, C + G; W, A + T; Y, C + T. Protein names are as given Table 1. [file 1471-2148-12-127-S4.pdf]

Tab. S2: Oligonucleotide primers for cDNA isolation. Sequences are given starting with the 5' end. Mixed nucleotide codes are: D, A+G+T; K, G+T; M, A+C; R, A+G; S, C+G; W, A+T; Y, C+T. Protein names are as given Table 1.

Species /protein	Purpose	Name	Nucleotide sequence
all	degenerate	P1	TCG ACA AAS ACC TYT ACG TCT TTG ACT TCA A
all	degenerate	P2	TCC CAC ACC ART GTC TCR GTG TCC AA
all	degenerate	P5	TCT ATD YCT WYG GWG GCC GDG AC
all	degenerate	P6	TCA TTG STY KGM GMD TTM CCR CCA
all	oligo(dT)anchor	P7	GGC CAC GCG TCG ACT AGT ACT TTT TTT TTT TTT TTT TT
ApTFP1	3´RACE	P54	ATC CTA ATA TAC CAA CAC TC
ApTFP1	3´RACE/nested	P53	GCA CAT CAA GGC CCG GGG ACC
ApTFP1	5´RACE	P51	TCG GGT CCT CCC TCT TCA TC
ApTFP1	5´RACE/nested	P50	CCC TTG GCC GGA GCG ATT GAC C
ChESP1	3´RACE	P12	CCC GGG ACG TTG AAC AAT GAG GG
ChESP1	3´RACE/nested	P11	GAG GGT TAT GTG TTG GAC AC
ChESP1	5´RACE	P10	GGG GCT TCT CCG TTG GCC GGA GCG
ChESP1	5´RACE/nested	P9	CCA AGT CTG AGT GTT GAA GTC
ChESP1	ORF	P13	GGC TTA AUA TGG CTC CAA CTT TTC AAG GCG
ChESP1	ORF	P14	GGT TTA AUT TAA GCT GAG TTA ACA GCG T
ChNSP1	3´RACE	P20	GGG ATC ATT GAT GGT AAG AAA GGG C
ChNSP1	3´RACE/nested	P19	GGG CTT GTG ATG CAT GGC GG
ChNSP1	5´RACE	P25	GCC GTT CCG TAC CAC CCA CTC C
ChNSP1	5´RACE/nested	P18	GTC GTG TCA AAC GAG TAG AAG CCG
ChNSP1	ORF	P28	GGC TTA AUA TGG CCC AAA AGC TGG AAG
ChNSP1	ORF	P29	GGT TTA AUC TAA TGG ATT CCA TAA AAG AAG
CiESP1	3´RACE	P76	GCG TGT TTG CGC ATG CCG TAG
CiESP1	3´RACE/nested	P12	CCC GGG ACG TTG AAC AAT GAG GG
CiESP1	5´RACE	P75	GGT CCT CCC GCC TCA TCG
CiESP1	5´RACE/nested	P74	GAG TAA AAG TCG CAG AAG TGG C
CiESP1	ORF	P13	GGC TTA AUA TGG CTC CAA CTT TTC AAG GCG
CiESP1	ORF	P14	GGT TTA AUT TAA GCT GAG TTA ACA GCG T
DaESP1	3´RACE	P68	CCC GGC TTC TGG AAA ATG GAC
DaESP1	3´RACE/nested	P67	CGA AGC CAG ACC CAA ATG CCC
DaESP1	5´RACE	P66	CGA GTA AAA GTC TTC GAA GTC C
DaESP1	5´RACE/nested	P65	CGG CCT CCA AAG ATA TAG AGC
DaESP1	ORF	P69	GGC TTA AUA TGG CTC CGA GTT TAC AAG G
DaESP1	ORF	P70	GGT TTA AUT TAC GCG GAA TTG ACT GCA TAG AAG
DlESP1	3´RACE	P68	CCC GGC TTC TGG AAA ATG GAC
DlESP1	3´RACE/nested	P67	CGA AGC CAG ACC CAA ATG CCC
DlESP1	5´RACE	P66	CGA GTA AAA GTC TTC GAA GTC C
DlESP1	5´RACE/nested	P65	CGG CCT CCA AAG ATA TAG AGC
DlESP1	ORF	P69	GGC TTA AUA TGG CTC CGA GTT TAC AAG G
DlESP1	ORF	P70	GGT TTA AUT TAC GCG GAA TTG ACT GCA TAG AAG
ItESP1	3´RACE	P128	CGT GTT CGC GCA TGC CGT TGT AGG
ItESP1	3´RACE/nested	P127	GGA GGT GAA ACA TGG CCG GAT CC
ItESP1	5´RACE	P114	GCG GCC TCC GAA GAG ATA GAG C
ItESP1	5´RACE	P115	CGA ACG AGT AAA ACT CGT CG
ItESP1	ORF	P130	GGC TTA AUA TGG CTC CGA GTG TGC AAG GC
ItESP1	ORF	P131	GGT TTA AUT TAC GCG GAA TTG ACT GCA TAG
ItNSP1	3´RACE	P142	GGA CAC AAG TGG AAA CAT ATG G
ItNSP1	3´RACE/nested	P141	GCC TTG TGC TAG GAG CGT TTT CG
ItNSP1	5´RACE	P145	GGT GGG AAG CGT CTC TGC CTA C
ItNSP1	5´RACE/nested	P144	CCA ACT GAG ACC ATA CGA ACA C
ItNSP1	ORF	P149	GGC TTA AUA TGG CCA AAA AGC TGG AAG CAA AGG
ItNSP1	ORF	P150	GGT TTA AUT TAA GCA GAG TCA ATC CCG
SpESP1	3´RACE	P115	CGA ACG AGT AAA ACT CGT CG
SpESP1	3´RACE/nested	P114	GCG GCC TCC GAA GAG ATA GAG C

SpESP1	5´RACE	P112	GAT CTG GCC GGA CCC AAA CGG TC
SpESP1	5´RACE/nested	P111	CGG TCC AGG GAC GTT GAC C
SpESP1	ORF	P118	GGC TTA AUA TGG CTT CGA CAT TGC AAG GC
SpESP1	ORF	P119	GGT TTA AUT TAC GCA GAA TTG ACA GCG TAG
SpNSP1	3´RACE	P99	GCC AGG AGT GTT TTC GCT AGT GC
SpNSP1	3´RACE/nested	P98	GGT AAA CAC ATT GTG ATA TTT GG
SpNSP1	5´RACE	P96	CTT GAA GCG TCA CGG CCT CC
SpNSP1	5´RACE/nested	P95	CCA ATA GAC ACC ATG CGG ACG C
SpNSP1	ORF	P102	GGC TTA AUA TGG CCC AAA AGC TGG AAG C
SpNSP1	ORF	P103	GGT TTA AUT TAA GCA GAT TCA ATC CCA TAA
