# Supplementary material for: Evolution of specifier proteins in glucosinolate-containing plants
Source: BMC Evol Biol. 2012 Jul 28;12:127. doi: 10.1186/1471-2148-12-127 (PMC3482593; doi:10.1186/1471-2148-12-127)
Supplement: Additional file 5 — Figure S3. Assignment of newly identified specifier proteins to the ESP, TFP or NSP type. Activity was tested using crude extracts of bacteria expressing specifier protein cDNAs, 1 mM allylglucosinolate (A-K) or 2 mM benzylglucosinolate (L-V) and 0.005 units myrosinase in 50 mM MES buffer, pH 6.0. Shown are total ion current traces of GC-MS chromatograms recorded of dichloromethane extracts of the assay mixtures after 30 min incubation. Products of allylglucosinolate breakdown are simple nitrile (1), organic thiocyanate (2), isothiocyanate (3), and epithionitrile (4). Products of benzylglucosinolate are simple nitrile (5), and isothiocyanate (6). (X) indicates the peak of the internal standard. The following cDNAs were used: A, L, no cDNA (empty vector control); B, M, ApTFP; C, N, ChESP; D, O, CiESP; E, P, DaESP; F, Q, DlESP; G, R, ItESP; H, S, SpESP; I, T, ChNSP; J, U, ItNSP; K, V, SpNSP. [file 1471-2148-12-127-S5.pdf]

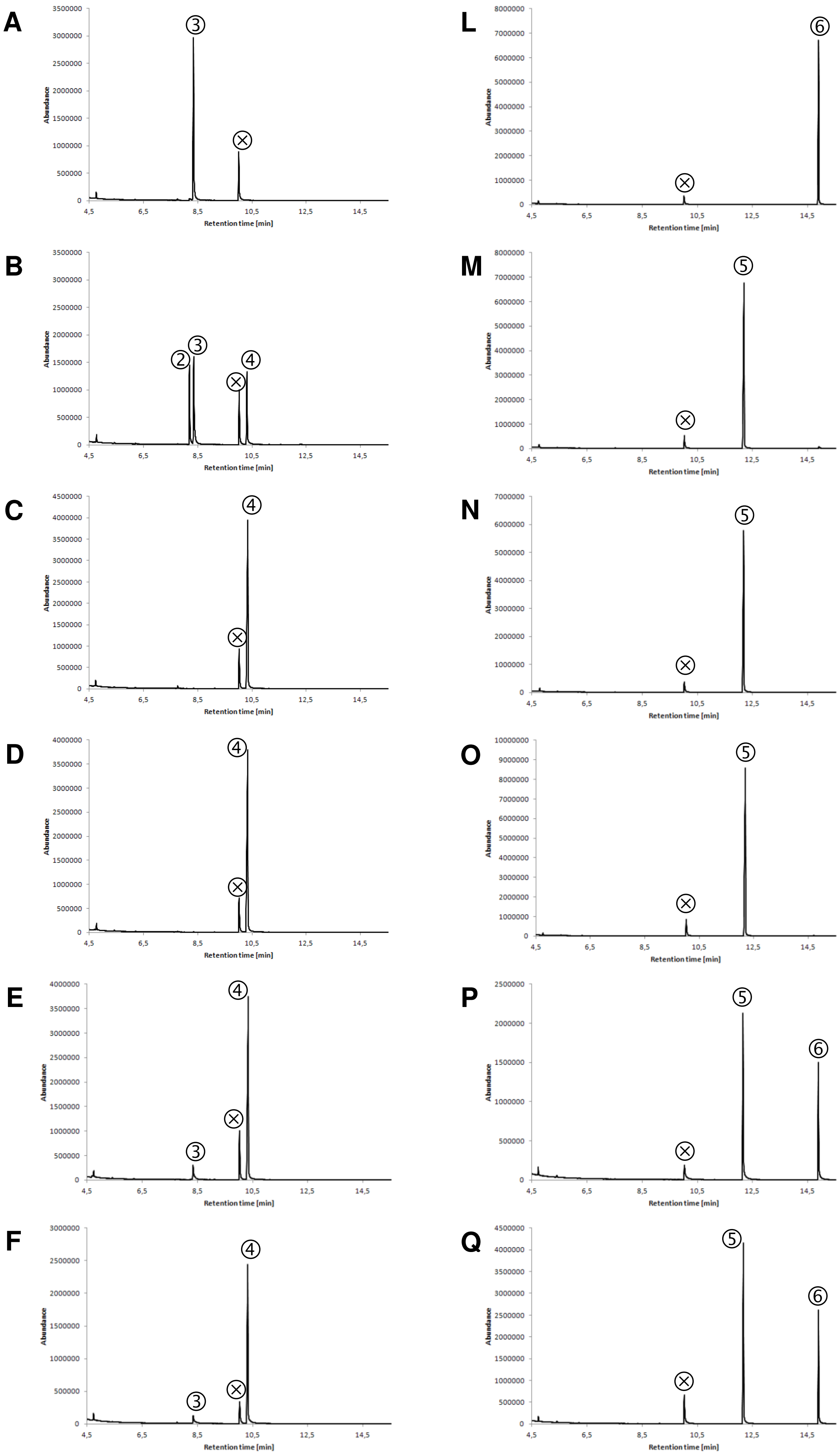

A
B
C
D
E
F
L
M
N
O
P
Q
Abundance
Retention time [min]
4,5
6,5
8,5
10,5
12,5
14,5
0
500000
1000000
1500000
2000000
2500000
3000000
3500000
4000000
4500000
5000000
6000000
7000000
8000000
9000000
10000000

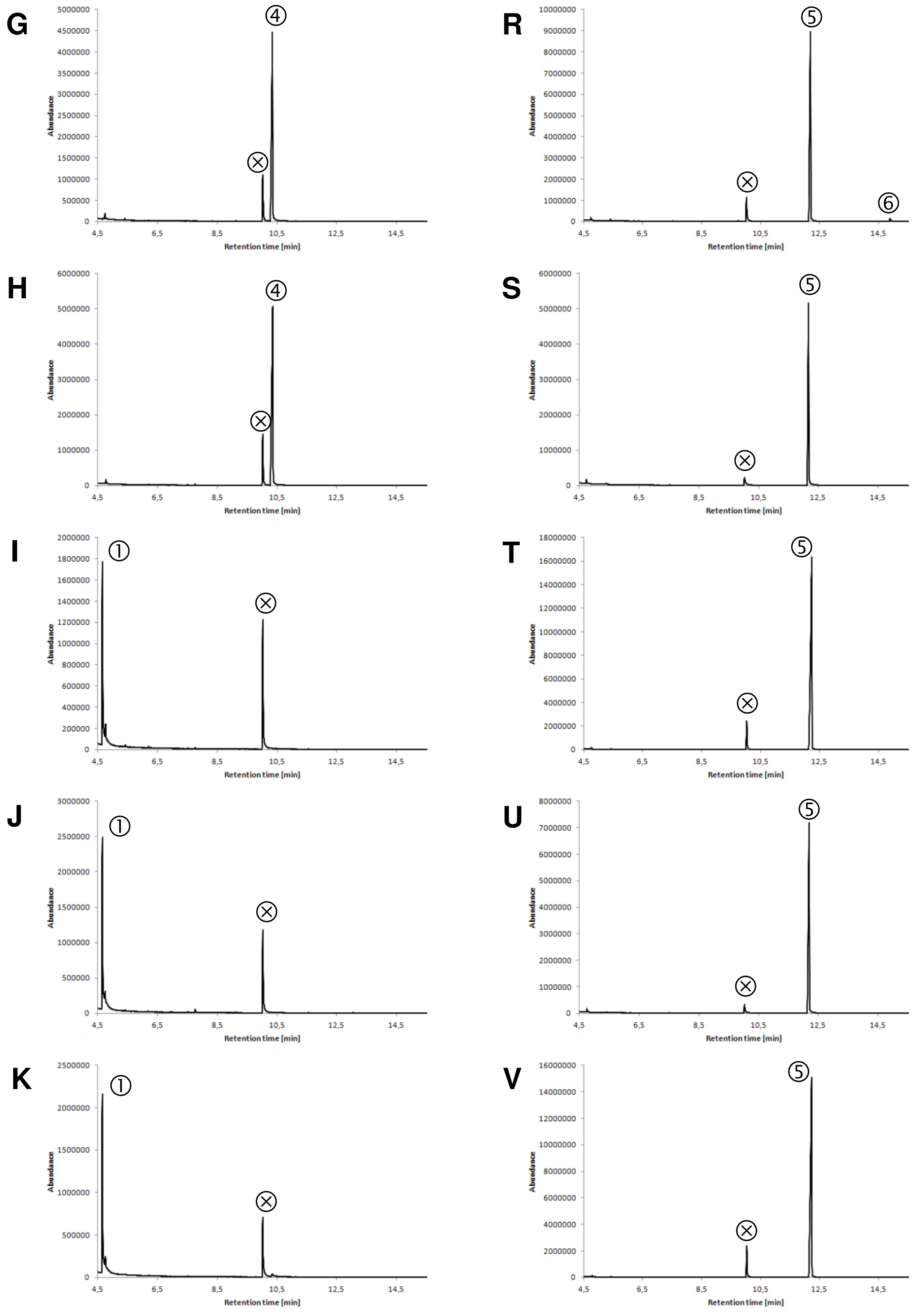

Fig. S3: Assignment of newly identified specifier proteins to the ESP, TFP or NSP type. Activity was tested using crude extracts of bacteria expressing specifier protein cDNAs, 1 mM allylglucosinolate (**A-K**) or 2 mM benzylglucosinolate (**L-V**) and 0.005 units myrosinase in 50 mM MES buffer, pH 6.0. Shown are total ion current traces of GC-MS chromatograms recorded of dichloromethane extracts of the assay mixtures after 30 min incubation. Products of allylglucosinolate breakdown are simple nitrile (1), organic thiocyanate (2), isothiocyanate (3), and epithionitrile (4). Products of benzylglucosinolate are simple nitrile (5), and isothiocyanate (6). (X) indicates the peak of the internal standard. The following cDNAs were used: **A, L**, no cDNA (empty vector control); **B, M**, *ApTFP*; **C, N**, *ChESP*; **D, O**, *CiESP*; **E, P**, *DaESP*; **F, Q**, *DlESP*; **G, R**, *ItESP*; **H, S**, *SpESP*; **I, T**, *ChNSP*; **J, U**, *ItNSP*; **K, V**, *SpNSP*.
