# Supplementary material for: Evolution of specifier proteins in glucosinolate-containing plants
Source: BMC Evol Biol. 2012 Jul 28;12:127. doi: 10.1186/1471-2148-12-127 (PMC3482593; doi:10.1186/1471-2148-12-127)
Supplement: Additional file 6 — Table S3. Amino acid and nucleotide sequence identities of specifier proteins/ specifier protein cDNAs. Amino acid sequence identities are shown on the right, nucleotide sequence identities on the left as determined in separate comparisons of each sequence pair by ClustalW implemented in MEGA Vers. 5.05. [file 1471-2148-12-127-S6.pdf]

Tab. S3: Amino acid and nucleotide sequence identities of specifier proteins/ specifier protein cDNAs. Amino acid sequence identities are shown on the right, nucleotide sequence identities on the left as determined in separate comparisons of each sequence pair by ClustalW implemented in MEGA Vers. 5.05.

	Lepidium sativum (Lepidieae) LsTFP	Alliaria petiolata (Thlaspideae) TFP1	Thlaspi arvense (Thlaspideae) TaTFP	Draba aurea (Arabideae) ESP1	Draba lanceolata (Arabideae) ESP1	Brassica oleracea (Brassiceae) ESP	Brassica rapa (Brassiceae) FJ374638	Schouwia purpurea (Brassiceae) ESP1	Arabidopsis lyrata (Camelineae) XM002884120	Arabidopsis thaliana (Camelineae) AtESP	Cardamine hirsuta (Cardamineae) ESP1	Cardamine impatiens (Cardamineae) ESP1	Isatis tinctoria (Isatideae) ESP1	Schouwia purpurea (Brassiceae) NSP1	Arabidopsis thaliana (Camelineae) AtNSP1	Arabidopsis thaliana (Camelineae) AtNSP2	Arabidopsis thaliana (Camelineae) AtNSP3	Arabidopsis thaliana (Camelineae) AtNSP4	Arabidopsis thaliana (Camelineae) AtNSP5	Arabidopsis thaliana (Camelineae) At3g07720	Cardamine hirsuta (Cardamineae) NSP1	Eutrema halophilum (Eutremeae) AK353179	Isatis tinctoria (Isatideae) NSP1
Lepidium sativum (Lepidieae) LsTFP		60	59	67	67	63	64	65	67	68	65	64	63	51	51	49	51	48	46	44	49	45	48
Alliaria petiolata (Thlaspideae) TFP1	73		93	74	73	75	75	76	72	72	68	67	76	53	53	54	52	50	46	47	52	46	52
Thlaspi arvense (Thlaspideae) TaTFP	73	94		72	72	72	73	73	70	70	67	65	75	52	52	63	51	50	46	46	51	47	52
Draba aurea (Arabideae) ESP1	75	80	79		98	79	76	78	78	79	74	72	81	57	57	57	56	55	47	49	55	48	55
Draba lanceolata (Arabideae) ESP1	75	80	80	98		80	76	78	78	78	74	72	81	58	56	57	56	55	47	48	54	48	55
Brassica oleracea (Brassiceae) ESP	74	80	79	81	81		85	89	76	77	73	71	89	56	55	57	54	53	48	47	53	48	54
Brassica rapa (Brassiceae) FJ374638	76	82	81	80	80	87		85	77	76	75	72	84	53	55	54	53	53	47	48	53	47	53
Schouwia purpurea (Brassiceae) ESP1	77	82	82	82	82	88	87		76	78	73	71	86	56	57	56	55	54	46	49	54	48	55
Arabidopsis lyrata (Camelineae) XM002884120	78	78	78	80	80	79	81	81		83	78	76	79	57	55	57	54	53	47	46	53	49	55
Arabidopsis thaliana (Camelineae) AtESP	78	79	78	81	81	80	81	81	85		78	76	78	56	56	56	55	54	50	48	53	51	54
Cardamine hirsuta (Cardamineae) ESP1	77	77	76	79	79	79	80	80	83	82		85	76	54	52	53	51	50	48	47	51	48	51
Cardamine impatiens (Cardamineae) ESP1	74	77	76	78	79	78	79	80	83	83	89		73	54	53	54	51	51	48	47	51	49	52
Isatis tinctoria (Isatideae) ESP1	75	83	83	84	84	88	87	88	81	82	80	81		58	57	60	57	55	48	48	55	49	57
Schouwia purpurea (Brassiceae) NSP1	67	65	64	68	69	66	65	68	68	65	67	66	65		80	80	79	77	53	52	81	54	83
Arabidopsis thaliana (Camelineae) AtNSP1	67	63	63	67	67	66	64	67	66	66	64	64	66	85		81	90	92	53	51	84	53	77
Arabidopsis thaliana (Camelineae) AtNSP2	64	66	66	68	68	64	66	66	67	66	65	64	65	85	86		79	79	52	52	82	54	75
Arabidopsis thaliana (Camelineae) AtNSP3	68	63	64	66	67	63	64	65	67	67	64	63	65	83	92	85		85	50	49	81	52	74
Arabidopsis thaliana (Camelineae) AtNSP4	67	64	64	67	68	68	66	66	67	67	65	65	67	83	95	85	90		52	50	81	52	74
Arabidopsis thaliana (Camelineae) AtNSP5	63	63	63	66	65	64	63	61	64	64	64	61	64	67	67	68	66	67		54	51	85	52
Arabidopsis thaliana (Camelineae) At3g07720	62	62	63	61	61	64	62	65	63	64	65	64	62	67	66	66	66	66	65		51	57	51
Cardamine hirsuta (Cardamineae) NSP1	65	63	62	67	67	65	65	65	68	67	65	64	66	84	88	86	86	87	66	66		51	75
Eutrema halophilum (Eutremeae) AK353179	62	62	64	64	65	66	65	63	63	63	65	61	62	68	67	68	67	70	84	65	66		53
Isatis tinctoria (Isatideae) NSP1	65	65	64	67	66	66	65	67	67	67	67	65	65	87	83	82	82	82	67	67	83	68	
