# Supplementary material for: Evolution of specifier proteins in glucosinolate-containing plants
Source: BMC Evol Biol. 2012 Jul 28;12:127. doi: 10.1186/1471-2148-12-127 (PMC3482593; doi:10.1186/1471-2148-12-127)
Supplement: Additional file 7 — Figure S4. Phylogenetic tree of specifier proteins from Brassicaceae. Full-length amino acid sequences of 19 biochemically characterized NSPs, ESPs and TFPs (Table 1) as well as three putative specifier proteins (Table 3) and one homolog of unknown function (At3g07720) were subjected to phylogenetic analysis using the Maximum Likelihood algorithm with 1000 bootstrap repetitions. [file 1471-2148-12-127-S7.pdf]

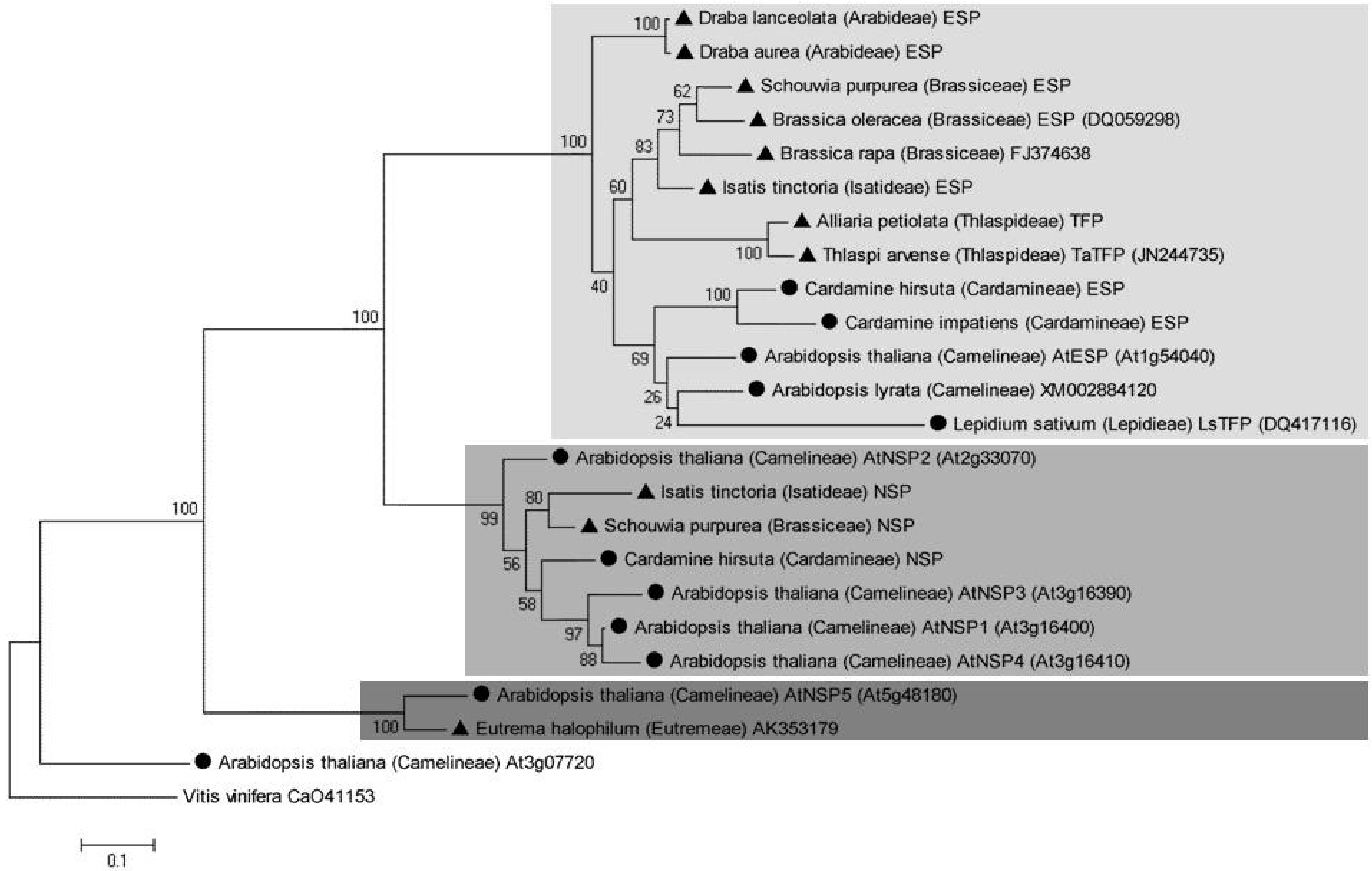

Fig. S4: Phylogenetic tree of specifier proteins from Brassicaceae. Full-length amino acid sequences of 19 biochemically characterized NSPs, ESPs and TFPs (Tab. 1) as well as three putative specifier proteins (Tab. 3) and one homolog of unknown function (At3g07720) were subjected to phylogenetic analysis using the Maximum Likelihood algorithm with 1000 bootstrap repetitions. Bootstrap values are given at the nodes. A homolog from *Vitis vinifera* (Vitaceae) which does not contain glucosinolates was used as an outgroup. Alignment gaps (e.g. JAL domains that are present only in NSPs) are regarded as non-informative posititions in this analysis. Symbols indicate Brassicaceae lineages (lineage I, circle; expanded lineage II, triangle). Boxes indicate clusters (dark grey, AtNSP5 cluster; grey, AtNSP1 cluster; light grey, ESP/TFP cluster). Branch lengths refer to the number of substitutions per site. A scale bar is given below the tree.
