# Supplementary material for: Evolution of specifier proteins in glucosinolate-containing plants
Source: BMC Evol Biol. 2012 Jul 28;12:127. doi: 10.1186/1471-2148-12-127 (PMC3482593; doi:10.1186/1471-2148-12-127)
Supplement: Additional file 9 — Figure S6. Maximum Parsimony tree of specifier protein cDNAs from Brassicaceae. Full-length nucleotide sequences of 19 biochemically characterized NSPs, ESPs and TFPs (Table 1) as well as three putative specifier proteins (Table 3) and one homolog of unknown function (At3g07720) were subjected to phylogenetic analysis using the Maximum Parsimony algorithm with 1000 bootstrap repetitions. Bootstrap values are given at the nodes. A homolog from Vitis vinifera (Vitaceae) which does not contain glucosinolates was used as an outgroup. Alignment gaps (e.g. JAL domains that are present only in NSPs) are regarded as non-informative posititions in this analysis. [file 1471-2148-12-127-S9.pdf]

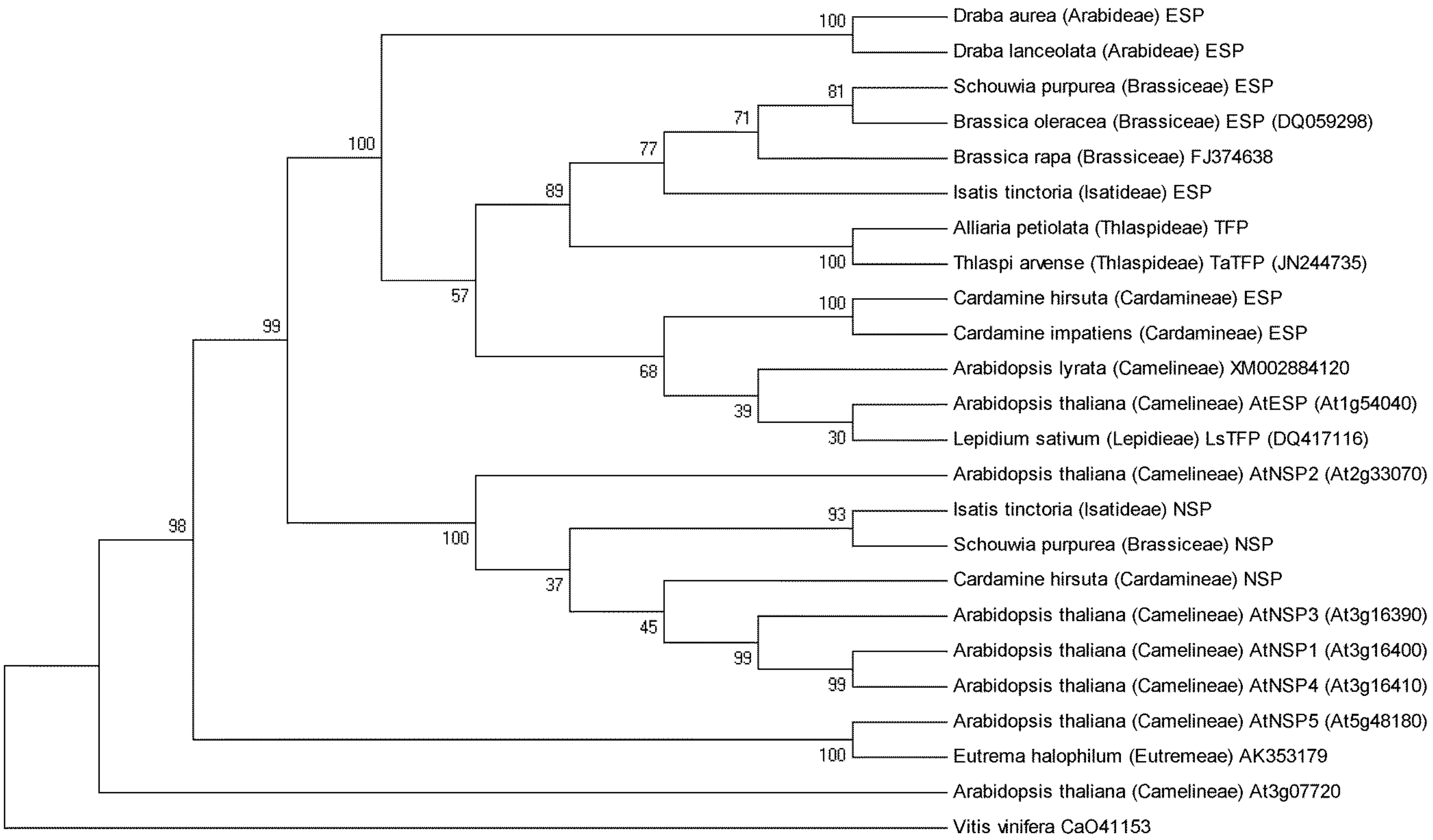

Fig. S5 (pdf): Maximum Parsimony tree of specifier protein cDNAs from Brassicaceae. Full-length nucleotide sequences of 19 biochemically characterized NSPs, ESPs and TFPs (Tab. 1) as well as three putative specifier proteins (Tab. 3) and one homolog of unknown function (At3g07720) were subjected to phylogenetic analysis using the Maximum Parsimony algorithm with 1000 bootstrap repetitions. Bootstrap values are given at the nodes. A homolog from *Vitis vinifera* (Vitaceae) which does not contain glucosinolates was used as an outgroup. Alignment gaps (e.g. JAL domains that are present only in NSPs) are regarded as non-informative posititions in this analysis.
