# Supplementary material for: Evolution of specifier proteins in glucosinolate-containing plants
Source: BMC Evol Biol. 2012 Jul 28;12:127. doi: 10.1186/1471-2148-12-127 (PMC3482593; doi:10.1186/1471-2148-12-127)
Supplement: Additional file 10 — Table S4. Amino acid and nucleotide distances of specifier proteins/ specifier protein cDNAs. Amino acid distances are shown on the right, nucleotide distances on the left as determined by MEGA Vers. 5.05 using the Equal-input + G model (daa) and the Kimura-Two-Parameter model + G + I (dna). Alignment gaps (e.g. JAL domains that are present only in NSPs) are regarded as non-informative posititions in this analysis. [file 1471-2148-12-127-S10.pdf]

Tab. S4: Amino acid and nucleotide distances of specifier proteins. Amino acid distances are shown on the right, nucleotide distances on the left as determined by MEGA Vers. 5.05 using the Equal-input +G model (d_{aa}) and the Kimura-Two-Parameter model +G +I (d_{na}). Alignment gaps (e.g. JAL domains that are present only in NSPs) are regarded as non-informative posititions in this analysis.

	Lepidium sativum (Lepidieae) LsTFP	Alliaria petiolata (Thlaspideae) TFP1	Thlaspi arvense (Thlaspideae) TaTFP	Draba aurea (Arabideae) ESP1	Draba lanceolata (Arabideae) ESP1	Brassica oleraceae (Brassiceae) ESP	Brassica rapa (Brassiceae) FJ374638	Schouwia purpurea (Brassiceae) ESP1	Arabidopsis lyrata (Camelineae) XM002884120	Arabidopsis thaliana (Camelineae) AtESP	Cardamine hirsuta (Cardamineae) ESP1	Cardamine impatiens (Cardamineae) ESP1	Isatis tinctoria (Isatideae) ESP1	Schouwia purpurea (Brassiceae) NSP1	Arabidopsis thaliana (Camelineae) AtNSP1	Arabidopsis thaliana (Camelineae) AtNSP2	Arabidopsis thaliana (Camelineae) AtNSP3	Arabidopsis thaliana (Camelineae) AtNSP4	Arabidopsis thaliana (Camelineae) AtNSP5	Arabidopsis thaliana (Camelineae) At3g07720	Cardamine hirsuta (Cardamineae) NSP1	Eutrema halophilum (Eutremeae) AK353179	Isatis tinctoria (Isatideae) NSP1
Lepidium sativum (Lepidieae) LsTFP		0,681	0,701	0,503	0,495	0,605	0,578	0,561	0,497	0,479	0,560	0,605	0,587	1,010	1,024	1,071	0,985	1,151	1,461	1,461	1,102	1,508	1,136
Alliaria petiolata (Thlaspideae) TFP1	0,531		0,065	0,335	0,341	0,323	0,317	0,299	0,374	0,373	0,434	0,495	0,282	0,812	0,813	0,790	0,815	0,926	1,336	1,316	0,861	1,399	0,838
Thlaspi arvense (Thlaspideae) TaTFP	0,545	0,059		0,347	0,354	0,366	0,341	0,341	0,408	0,406	0,464	0,527	0,299	0,836	0,848	0,824	0,839	0,952	1,335	1,396	0,873	1,317	0,862
Draba aurea (Arabideae) ESP1	0,419	0,290	0,303		0,010	0,250	0,294	0,266	0,272	0,266	0,348	0,393	0,219	0,713	0,693	0,693	0,704	0,756	1,262	1,172	0,757	1,264	0,815
Draba lanceolata (Arabideae) ESP1	0,416	0,292	0,305	0,008		0,244	0,294	0,266	0,267	0,266	0,335	0,380	0,219	0,703	0,713	0,693	0,694	0,779	1,243	1,207	0,779	1,263	0,815
Brassica oleraceae (Brassiceae) ESP	0,445	0,274	0,291	0,271	0,270		0,185	0,111	0,312	0,294	0,354	0,414	0,123	0,735	0,724	0,703	0,747	0,802	1,207	1,281	0,826	1,282	0,827
Brassica rapa (Brassiceae) FJ374638	0,439	0,234	0,252	0,275	0,274	0,169		0,166	0,295	0,299	0,311	0,380	0,180	0,825	0,734	0,790	0,780	0,813	1,242	1,171	0,813	1,359	0,876
Schouwia purpurea (Brassiceae) ESP1	0,400	0,232	0,242	0,234	0,229	0,124	0,155		0,312	0,271	0,354	0,407	0,153	0,745	0,682	0,703	0,725	0,768	1,319	1,138	0,779	1,301	0,791
Arabidopsis lyrata (Camelineae) XM002884120	0,353	0,302	0,332	0,268	0,262	0,295	0,254	0,254		0,200	0,278	0,312	0,245	0,749	0,818	0,717	0,808	0,905	1,232	1,347	0,867	1,215	0,831
Arabidopsis thaliana (Camelineae) AtESP	0,345	0,309	0,333	0,246	0,245	0,279	0,258	0,234	0,184		0,282	0,311	0,266	0,790	0,779	0,756	0,781	0,850	1,073	1,206	0,850	1,090	0,814
Cardamine hirsuta (Cardamineae) ESP1	0,396	0,360	0,381	0,299	0,295	0,293	0,271	0,256	0,219	0,248		0,162	0,299	0,824	0,900	0,862	0,890	0,981	1,187	1,278	0,940	1,242	0,941
Cardamine impatiens (Cardamineae) ESP1	0,408	0,357	0,389	0,303	0,299	0,319	0,285	0,255	0,216	0,213	0,125		0,354	0,837	0,887	0,850	0,904	0,982	1,188	1,260	0,954	1,190	0,955
Isatis tinctoria (Isatideae) ESP1	0,411	0,196	0,222	0,213	0,211	0,132	0,141	0,120	0,230	0,228	0,257	0,248		0,662	0,662	0,605	0,663	0,745	1,206	1,206	0,713	1,244	0,724
Schouwia purpurea (Brassiceae) NSP1	0,875	0,734	0,764	0,665	0,651	0,697	0,730	0,637	0,702	0,749	0,686	0,787	0,676		0,185	0,157	0,201	0,234	0,955	0,983	0,153	0,956	0,153
Arabidopsis thaliana (Camelineae) AtNSP1	0,853	0,772	0,796	0,653	0,653	0,711	0,696	0,656	0,730	0,761	0,690	0,812	0,659	0,166		0,194	0,100	0,057	0,983	1,012	0,162	0,984	0,229
Arabidopsis thaliana (Camelineae) AtNSP2	0,957	0,707	0,730	0,666	0,673	0,741	0,699	0,679	0,744	0,744	0,711	0,807	0,690	0,157	0,145		0,232	0,229	1,012	0,969	0,180	0,956	0,239
Arabidopsis thaliana (Camelineae) AtNSP3	0,888	0,729	0,752	0,662	0,657	0,762	0,733	0,692	0,717	0,772	0,709	0,825	0,664	0,181	0,085	0,160		0,155	1,047	1,095	0,216	1,033	0,286
Arabidopsis thaliana (Camelineae) AtNSP4	0,889	0,800	0,840	0,667	0,667	0,739	0,730	0,687	0,769	0,788	0,727	0,849	0,683	0,191	0,041	0,155	0,108		1,026	1,104	0,214	1,012	0,271
Arabidopsis thaliana (Camelineae) AtNSP5	1,159	1,189	1,168	0,971	0,994	1,045	1,044	1,061	1,040	1,034	1,054	1,153	1,023	0,886	0,818	0,827	0,893	0,838		0,928	1,073	0,149	0,998
Arabidopsis thaliana (Camelineae) At3g07720	1,211	1,230	1,205	0,971	0,996	1,018	1,046	0,943	0,982	1,090	1,061	1,086	1,083	0,815	0,800	0,783	0,844	0,845	0,872		1,012	0,827	1,028
Cardamine hirsuta (Cardamineae) NSP1	0,935	0,757	0,790	0,662	0,665	0,760	0,729	0,703	0,725	0,760	0,751	0,890	0,687	0,177	0,127	0,154	0,155	0,140	0,828	0,820		1,074	0,229
Eutrema halophilum (Eutremeae) AK353179	1,238	1,178	1,096	1,040	1,033	1,066	1,081	1,076	1,039	1,077	1,031	1,126	1,078	0,864	0,864	0,794	0,894	0,920	0,215	0,853	0,852		0,971
Isatis tinctoria (Isatideae) NSP1	1,014	0,748	0,767	0,728	0,740	0,733	0,768	0,692	0,744	0,786	0,743	0,848	0,708	0,138	0,188	0,205	0,212	0,211	0,901	0,812	0,193	0,891	
