# Supplementary material for: Evolution of specifier proteins in glucosinolate-containing plants
Source: BMC Evol Biol. 2012 Jul 28;12:127. doi: 10.1186/1471-2148-12-127 (PMC3482593; doi:10.1186/1471-2148-12-127)
Supplement: Additional file 11 — Table S5. Non-synonymous vs. synonymous substitution rate ratios of specifier protein cDNAs.dN and dS were calculated by MEGA 5.05 from pairwise comparisons based on the Kimura-2-parameter model and used to determine ω as the dN / dS ratio. [file 1471-2148-12-127-S11.pdf]

Tab. S5: Non-synonymous vs. synonymous substitution rate ratios of specifier protein cDNAs. d_N and d_S were calculated by MEGA 5.05 from pairwise comparisons based on the Kimura-2-parameter model and used to determine ω as the d_N / d_S ratio.

	Lepidium sativum (Lepidieae) LsTFP	Alliaria petiolata (Thlaspideae) TFP1	Thlaspi arvense (Thlaspideae) TaTFP	Draba aurea (Arabideae) ESP1	Draba lanceolata (Arabideae) ESP1	Brassica oleraceae (Brassiceae) ESP	Brassica rapa (Brassiceae) FJ374638	Schouwia purpurea (Brassiceae) ESP1	Arabidopsis lyrata (Camelineae) XM002884120	Arabidopsis thaliana (Camelineae) AtESP	Cardamine hirsuta (Cardamineae) ESP1	Cardamine impatiens (Cardamineae) ESP1	Isatis tinctoria (Isatideae) ESP1	Schouwia purpurea (Brassiceae) NSP1	Arabidopsis thaliana (Camelineae) AtNSP1	Arabidopsis thaliana (Camelineae) AtNSP2	Arabidopsis thaliana (Camelineae) AtNSP3	Arabidopsis thaliana (Camelineae) AtNSP4	Arabidopsis thaliana (Camelineae) AtNSP5	Arabidopsis thaliana (Camelineae) At3g07720	Cardamine hirsuta (Cardamineae) NSP1	Eutrema halophilum (Eutremeae) AK353179
Alliaria petiolata (Thlaspideae) TFP1	0,516																					
Thlaspi arvense (Thlaspideae) TaTFP	0,524	0,198																				
Draba aurea (Arabideae) ESP1	0,380	0,348	0,344																			
Draba lanceolata (Arabideae) ESP1	0,385	0,352	0,352	0,627																		
Brassica oleraceae (Brassiceae) ESP	0,397	0,359	0,392	0,218	0,214																	
Brassica rapa (Brassiceae) FJ374638	0,412	0,403	0,381	0,277	0,265	0,213																
Schouwia purpurea (Brassiceae) ESP1	0,460	0,446	0,478	0,285	0,293	0,198	0,222															
Arabidopsis lyrata (Camelineae) XM002884120	0,427	0,444	0,415	0,279	0,285	0,304	0,339	0,360														
Arabidopsis thaliana (Camelineae) AtESP	0,449	0,383	0,377	0,287	0,297	0,265	0,305	0,285	0,258													
Cardamine hirsuta (Cardamineae) ESP1	0,405	0,428	0,417	0,379	0,364	0,321	0,281	0,388	0,322	0,325												
Cardamine impatiens (Cardamineae) ESP1	0,495	0,549	0,511	0,478	0,475	0,381	0,416	0,549	0,434	0,478	0,394											
Isatis tinctoria (Isatideae) ESP1	0,448	0,502	0,439	0,273	0,269	0,143	0,265	0,287	0,331	0,327	0,341	0,522										
Schouwia purpurea (Brassiceae) NSP1	0,312	0,312	0,333	0,240	0,241	0,250	0,276	0,299	0,264	0,244	0,278	0,253	0,236									
Arabidopsis thaliana (Camelineae) AtNSP1	0,309	0,306	0,308	0,248	0,254	0,233	0,280	0,262	0,277	0,242	0,294	0,260	0,253	0,300								
Arabidopsis thaliana (Camelineae) AtNSP2	0,251	0,320	0,342	0,262	0,254	0,225	0,271	0,245	0,236	0,238	0,293	0,251	0,219	0,288	0,356							
Arabidopsis thaliana (Camelineae) AtNSP3	0,295	0,342	0,346	0,250	0,249	0,236	0,281	0,277	0,294	0,250	0,302	0,285	0,278	0,291	0,308	0,351						
Arabidopsis thaliana (Camelineae) AtNSP4	0,335	0,321	0,323	0,255	0,261	0,253	0,289	0,282	0,282	0,246	0,307	0,279	0,270	0,316	0,319	0,349	0,418					
Arabidopsis thaliana (Camelineae) AtNSP5	0,312	0,250	0,238	0,328	0,326	0,272	0,252	0,297	0,269	0,222	0,272	0,262	0,292	0,239	0,300	0,289	0,307	0,296				
Arabidopsis thaliana (Camelineae) At3g07720	0,244	0,234	0,288	0,223	0,240	0,316	0,275	0,302	0,351	0,239	0,311	0,269	0,236	0,306	0,303	0,295	0,323	0,308	0,270			
Cardamine hirsuta (Cardamineae) NSP1	0,302	0,328	0,335	0,259	0,264	0,256	0,298	0,271	0,303	0,264	0,316	0,270	0,251	0,259	0,343	0,344	0,429	0,435	0,322	0,330		
Eutrema halophilum (Eutremeae) AK353179	0,269	0,287	0,278	0,276	0,285	0,290	0,270	0,296	0,281	0,225	0,315	0,263	0,273	0,294	0,269	0,300	0,305	0,255	0,123	0,256	0,319	
Isatis tinctoria (Isatideae) NSP1	0,281	0,306	0,332	0,255	0,246	0,281	0,296	0,292	0,271	0,231	0,312	0,279	0,266	0,360	0,324	0,348	0,383	0,357	0,277	0,298	0,372	0,302
