# Supplementary material for: Evolution of specifier proteins in glucosinolate-containing plants
Source: BMC Evol Biol. 2012 Jul 28;12:127. doi: 10.1186/1471-2148-12-127 (PMC3482593; doi:10.1186/1471-2148-12-127)
Supplement: Additional file 12 — Table S6. Sources of seeds for growing plants for phytochemical analysis and cDNA isolation. [file 1471-2148-12-127-S12.pdf]

Tab. S6: Sources of seeds for growing plants for phytochemical analysis and cDNA isolation.

Species	Source
Alliaria petiolata	Collected locally (Lahstedt, Germany)
Alyssum alpestre	Österr. Gartenbaugesellschaft Graz
Arabis glabra	Botanical Garden & Rhododendron Parc Bremen, Germany
Barbarea intermedia	Botanical Garden & Rhododendron Parc Bremen, Germany
Barbarea stricta	Botanical Garden & Rhododendron Parc Bremen, Germany
Barbarea vulgaris	Botanical Garden & Rhododendron Parc Bremen, Germany
Camelina microcarpa	Botanical Garden & Rhododendron Parc Bremen, Germany
Capsella bursa-pastoris	Medicinal Plant Garden, Technische Universität Braunschweig, Germany
Capsella rubella	Markus Piotrowski, Ruhr-Universität Bochum, Germany
Cardamine hirsuta	Botanical Garden & Rhododendron Parc Bremen, Germany
Cardamine impatiens	Botanical Garden Tübingen, Germany
Cleome hassleriana	Universität Hohenheim/Stuttgart
Cleome spinosa	Private garden (J.C. Kuchernig, Lahstedt, Germany)
Diplotaxis muralis	Botanical Garden & Rhododendron Parc Bremen, Germany
Draba aurea	Botanical Garden Bonn, Germany
Draba lanceolata	Botanical Garden Bonn, Germany
Draba muralis	Botanical Garden & Rhododendron Parc Bremen, Germany
Eruca sativa	Medicinal Plant Garden, Technische Universität Braunschweig, Germany
Erysimum cheiri	Botanical Garden Caen, France
Erysimum hieraciifolium	Botanical Garden & Rhododendron Parc Bremen, Germany
Iberis amara	Botanical Garden Krefeld, Germany
Isatis tinctoria	Medicinal Plant Garden, Technische Universität Braunschweig, Germany
Limnanthes douglasii	Botanical Garden Bonn, Germany
Moringa oleifera	Botanical Garden Nemmara-Palakkad, India
Reseda lutea	Botanical Garden Krefeld, Germany
Reseda luteola	Botanical Garden & Rhododendron Parc Bremen, Germany
Schouwia purpurea	Medicinal Plant Garden, Technische Universität Braunschweig, Germany
Tropaeolum minus	Botanical Garden Gießen, Germany
